# A paradigm-level evaluation of deep learning architectures for plant pest and disease recognition under data-scarce field conditions

**DOI:** 10.3389/fpls.2026.1807927

**Published:** 2026-04-17

**Authors:** Liya Hu, Bowen Shi, Shiqi Hu, Mingxuan Song, Anhao Yang, Zhengwen Wang, Juntao Yang, Guiying Tang, Bo Bai, Shubin Wang

**Affiliations:** 1 College of Geodesy and Geomatics, Shandong University of Science and Technology, Qingdao, China; 2 Shandong Provincial Key Laboratory of Field Crop Physiology, Ecology, and Efficient Production (Under Construction), Institute of Crop Germplasm Resources, Shandong Academy of Agricultural Sciences, Jinan, Shandong, China; 3 Institute of Vegetables, Shandong Academy of Agricultural Sciences, Jinan, China

**Keywords:** agricultural vision applications, deep learning, pathology-oriented image analysis, plant disease phenotyping, plant pest and disease recognition

## Abstract

**Introduction:**

Accurate recognition of plant pests and diseases under field conditions remains challenging due to complex symptom morphology, environmental variability, and limited annotated data. While deep learning has been widely adopted for image-based diagnosis, existing studies are often model-centric and evaluated under heterogeneous experimental settings, making it difficult to derive paradigm-level insights into data efficiency, robustness, and practical deployment suitability.

**Methods:**

In this research, we systematically compare three main types of vision models: Convolutional Neural Networks (CNNs), Vision Transformers, and mixed State Space Model-based structures (MambaVision). These models are applied to classify images of pests and diseases across multiple crops. Using a unified and reproducible experimental framework, we benchmark representative models across multiple training regimes, diverse crop species, and symptom types reflecting realistic agricultural scenarios.

**Results:**

Results reveal clear paradigm-level differences. CNN-based models perform competitively on diseases dominated by localized lesion textures but show limited robustness for symptoms requiring global spatial interpretation. Transformer-based models benefit from global dependency modeling yet exhibit increased instability under small-sample conditions. In contrast, hybrid MambaVision-based models consistently demonstrate superior data efficiency and robustness, retaining approximately 60–80% accuracy under extreme data scarcity (1% training samples) and achieving stable, high F1-scores across symptom types that require joint modeling of fine-grained textures and long-range spatial distribution. Furthermore, performance–efficiency analysis shows that hybrid MambaVision-based models achieve a more favorable accuracy–computational cost trade-off than CNN-based and Transformer-based models, supporting deployment under practical resource constraints.

**Discussion:**

Overall, this study provides pathology-oriented and deployment-aware insights into how architectural inductive bias interacts with symptom morphology and data availability, highlighting hybrid MambaVision-based models as a robust and effective solution for real-world plant pest and disease recognition.

## 1 Introduction

Vegetable and field crops form the foundation of agricultural production systems and are essential for global food security and the stability of agri-food supply chains (Zhu et al., 2023b). Throughout their growth cycles, crops are exposed to biotic stresses such as insect pests and pathogenic diseases, which cause substantial yield losses and quality degradation, threatening the sustainability of agricultural systems (Zhang et al., 2022). Driven by climate change, intensified cultivation practices and expanded planting scales, the frequency and severity of pest and disease outbreaks have risen significantly, posing enduring challenges to modern crop management (Wang et al., 2023). Consequently, rapid and accurate diagnostic technologies for crop pest and disease identification are crucial for early hazard warning and sustainable agricultural development (Paul et al., 2025).

From the perspective of plant pathology, disease phenotypes are intrinsically complex, with symptoms featuring fine-grained lesion textures, irregular color variations and spatially heterogeneous distributions on leaf surfaces. Conventional diagnosis relying on manual field inspection is labor-intensive, subjective, and hard to scale, while remote sensing approaches face limitations in plant or leaf-level detection due to insufficient spatial resolution, spectral ambiguity and environmental interference (Nkwocha and Chandel, 2025; Radočaj et al., 2025; Jackulin and Murugavalli, 2022). Although laboratory-based chemical or spectral analyses can reach high diagnostic accuracy, their complex operation procedures, high costs, and poor deployability limit their routine application in field scenarios. These limitations highlight the need for diagnostic approaches that balance accuracy, efficiency, and scalability in actual agricultural settings (Rahman et al., 2025).

Plant pest and disease recognition differs fundamentally from generic object recognition tasks. Accurate diagnosis requires not only the detection of local visual cues like spots, mildew textures and necrotic regions, but also an understanding of their spatial distribution across the entire leaf surface. With the popularization of mobile devices and the advancement of deep learning technology, close-range image-based intelligent diagnosis has become a practical solution for plant-level pest and disease recognition (Batool and Byun, 2025). Such approaches enable non-destructive, low-cost, and real-time assessment under field conditions and are increasingly deployed in complex real-world agricultural scenarios (Batool and Byun, 2025; Shoaib et al., 2025). Nevertheless, designing effective deep learning models for agricultural image analysis remains a great challenge, especially under the constraints of limited annotated data, severe environmental interference and deployment-oriented computational efficiency (Bouacida et al., 2025).

Convolutional Neural Networks (CNNs) have been widely applied in crop pest and disease recognition due to their effectiveness in learning localized texture, color, and shape features (Wang et al., 2023). However, their limited receptive fields constrain global context modeling, reducing robustness under field conditions with uneven illumination, cluttered backgrounds, and spatially dispersed symptoms (Ahmad et al., 2023; Ali et al., 2024). Vision Transformers (ViTs) address this limitation by modeling long-range feature dependencies through self-attention mechanisms, but they bring significant memory and computational costs due to their quadratic complexity, which limits real-time deployment on resource-constrained devices and scalable applications to high-resolution agricultural imagery (Li et al., 2023; Xu et al., 2025). As a result, the suitability and trade-offs of different visual modeling paradigms for plant pest and disease recognition under realistic agricultural constraints remain insufficiently understood.

From the perspective of plant disease phenotyping, an effective visual recognition model needs to meet two core requirements. It should capture both fine-grained lesion characteristics and long-range spatial contextual information of leaves, while maintaining strong robustness to data scarcity and environmental noise. Hybrid architectures that integrate strong local feature representation with efficient global modeling have therefore gained increasing attention (Guo et al., 2025; Meng et al., 2025). Models like MambaVision integrate Transformer-style contextual modeling with the linear-time sequence processing efficiency of State Space Models (SSMs), achieving a favorable balance between representational capacity and computational efficiency (Mamun et al., 2025; Zhang et al., 2025). However, systematic comparative evaluations of CNN-, Transformer-, and hybrid SSM-based architectures in the task of plant pest and disease recognition are still lacking.

Despite the rapid progress of deep learning-based plant pest and disease recognition, existing studies are mostly model-centric and evaluated under heterogeneous datasets and experimental protocols, which makes it difficult to draw reliable paradigm-level conclusions about different visual architectures (Wang et al., 2025). In particular, key issues such as data efficiency under limited annotation, robustness to complex field conditions, and deployment-oriented performance-efficiency trade-offs remain insufficiently understood (Liu et al., 2025a; Mallick et al., 2025). Furthermore, most studies only report category-wise recognition accuracy without considering the morphological diversity of disease symptoms, and they overlook the interaction between architectural inductive bias and symptom characteristics, such as localized lesions and spatially distributed patterns. Although emerging hybrid architectures like MambaVision have shown great potential in general computer vision tasks, their effectiveness and robustness in agricultural pest and disease recognition, especially under severe data scarcity, have not yet been systematically investigated (Upadhyay et al., 2025; Mugisha et al., 2025).

To address these gaps, this study adopts a unified, paradigm-oriented evaluation framework to comparatively assess CNN-, Transformer-, and MambaVision-based models for multi-crop pest and disease image classification. Emphasis is placed on data efficiency, robustness, symptom-type dependency, and computational efficiency under realistic agricultural conditions. The main contributions are summarized as follows:

1. We establish a unified and reproducible benchmarking framework for systematic comparison of CNN-, Transformer-, and MambaVision-based models under consistent datasets, training protocols, and evaluation metrics, enabling fair cross-paradigm evaluation for agricultural pest and disease recognition.
2. Through multi-regime data efficiency experiments (1%–100% training data), we provide a comprehensive empirical analysis of paradigm-level differences in data efficiency and robustness, revealing the superior stability of MambaVision models under limited supervision.
3. We conduct fine-grained symptom-level and computational efficiency analyses, demonstrating that MambaVision models achieve a more favorable accuracy–efficiency trade-off than CNN and Transformer architectures, highlighting their potential for practical on-farm deployment under resource constraints.

## 2 Related works

Image-based plant pest and disease classification has been thoroughly studied with the rapid development of deep learning. However, from a plant pathology and phenotyping perspective, existing studies are highly fragmented in modeling paradigms and experimental settings. This makes it hard to draw paradigm-level conclusions about robustness, data efficiency and deployment feasibility under realistic agricultural conditions. Current approaches can be roughly grouped into three major paradigms: CNN-based models, Transformer-based vision models, and emerging hybrid MambaVision-based models. Each shows distinct advantages and limitations when applied to plant disease recognition tasks.

### 2.1 CNN-based methods for plant pest and disease classification

CNNs constitute the earliest and most widely adopted framework for plant pest and disease image classification. Their successful application mainly comes from strong local feature extraction capabilities, which fit well with capturing fine-grained disease features like lesions, color changes, mildew textures and edge irregularities on leaf surfaces. Representative studies have proved that deep CNNs can reach near-saturation performance on well-constructed datasets. For example, Mohanty et al. reported an accuracy of 99.35% on the PlantVillage dataset, validating the effectiveness of CNNs for leaf-level disease recognition (Mohanty et al., 2016). Ferentinos et al. further confirmed this conclusion by evaluating multiple CNN architectures on a large-scale dataset. The dataset includes more than 87,000 images covering 58 disease categories, with the model achieving a peak accuracy of 99.53% (Ferentinos, 2018). However, it should be noted that these results are obtained under specific dataset conditions, and may not directly generalize to more complex and heterogeneous datasets. This limitation highlights the need for further evaluation of model performance under more diverse and realistic data settings.

To alleviate data scarcity and improve generalization, transfer learning has been widely applied in agricultural scenarios. Chen et al. fine-tuned CNNs pretrained on ImageNet for plant disease identification under complex field conditions. They achieved an average accuracy of about 92.0% for several rice diseases, which demonstrates the effectiveness of transfer learning for medium and small-scale datasets (Chen et al., 2020). Complementing transfer learning strategies, recent architectural innovations have also sought to enhance feature extraction efficiency. For instance, a 2024 study introduced a Dense-Inception architecture integrated with attention modules specifically for plant disease imaging, demonstrating that such hybrid designs can significantly improve the discrimination of fine-grained lesion features in complex backgrounds (Khan et al., 2025).

Despite these research achievements, CNN-based models suffer from inherent limitations. Their receptive fields are fundamentally local, and modeling long-range spatial dependencies relies on deep stacking of convolutional layers. This leads to higher computational costs and greater optimization difficulty (Pei et al., 2025). Architectures such as Visual Geometry Group (VGG) and DenseNet perform well in feature representation learning, but bring huge memory and computation overhead, especially when processing high-resolution images. Although residual networks (e.g., ResNet and ResNeXt) mitigate vanishing gradients and enable deeper models, their ability to capture global se-mantic relationships remains limited.

These limitations show that CNN-based models, while effective in capturing local disease cues, struggle to stably characterize plant disease phenotypes. These phenotypes require joint analysis of fine-grained lesion details and global spatial organization, especially under data-limited field conditions (Pei et al., 2025; Salman et al., 2025; Tunio et al., 2024; Dhruw et al., 2025; Jia et al., 2025).

### 2.2 Transformer-based vision models

To overcome the local limitation of CNNs, ViTs and their variants introduce self-attention mechanisms. These mechanisms can explicitly model global contextual relationships across the entire image. This global modeling capability is especially beneficial for pest and disease recognition tasks involving spatially scattered lesions or multi-region symptoms. Recent studies have demonstrated the effectiveness of Transformer-based models in agricultural image analysis. For instance, Zhu et al. proposed MSCVT, which achieved a 99.86% test accuracy on the PlantVillage dataset with high precision, recall and F1-score. It also maintains a relatively compact parameter size (Zhu et al., 2023a).

In addition to disease classification, Transformer architectures have also been applied to plant disease detection and lightweight deployment scenarios. Wang et al. introduced PD-TR, an end-to-end Transformer-based framework for plant disease detection. It achieved a mean Average Precision (mAP) of 56.3 on a large-scale detection dataset, which highlights the challenges of real-world agricultural detection tasks (Wang et al., 2024). To facilitate mobile deployment, Li et al. developed a lightweight Vision Transformer. It achieved competitive accuracy on wheat, coffee and rice datasets with fewer than one million parameters, proving the feasibility of Transformer-based models on resource-constrained devices (Li et al., 2023). Beyond individual model development, systematic benchmarking has become a valuable approach to guide practical model selection in real-world agricultural settings. A 2025 benchmarking study focused on crop growth monitoring and weed detection in cotton fields, evaluating a suite of YOLO model variants to compare their performance under varying field conditions. This work underscored the critical need for such paradigm-level assessments, as they can better reveal a model’s true robustness and suitability for on-farm deployment (Raza et al., 2025).

However, Transformer-based models have obvious shortcomings. The quadratic computational complexity of self-attention with respect to spatial resolution leads to substantial overhead for high-resolution agricultural images (Saha and Xu, 2025). Moreover, Transformers usually need large-scale datasets and careful regularization strategies to achieve stable training. This makes them highly sensitive to data scarcity.

In plant disease recognition, this sensitivity to data availability and computational cost poses a critical challenge. Agricultural datasets are often limited in scale, and models must operate under deployment-oriented constraints (Fawole and Rawat, 2025).

### 2.3 Hybrid MambaVision-based models

Recently, SSMs have emerged as an efficient alternative for long-sequence modeling due to their linear computational complexity. Among them, the Mamba architecture introduces selective state-space mechanisms which enable efficient global dependency modeling (Mugisha et al., 2025). While Mamba has demonstrated strong performance in sequential domains, its original design targets one-dimensional sequences and lacks inductive biases suitable for two-dimensional visual data (Miao et al., 2025; Somvanshi et al., 2025).

To address this limitation, MambaVision integrates convolutional feature extraction with vision-adapted Mamba modules and Transformer-style components. It forms a hybrid architecture that balances local feature representation and global context modeling (Zhang et al., 2025). Convolutional layers capture fine-grained disease texture features, while Mamba-based state-space modules efficiently model long-range spatial dependencies with linear complexity. Self-attention blocks are further incorporated at the higher levels to enhance semantic information integration. This hybrid design shows that MambaVision-based architectures have the potential to balance fine-grained disease representation and efficient global context modeling. However, their relative advantages over CNN- and Transformer-based models in plant pest and disease recognition have not been fully verified via systematic comparative studies (Zhu et al., 2024).

In summary, existing studies show that CNN-based models are excellent at extracting local disease features but are limited by weak global modeling capability. Transformer-based models enhance global context perception, but at the cost of higher computational burden and greater sensitivity to data scarcity. Emerging hybrid MambaVision-based architectures provide a promising solution, by integrating convolutional inductive biases with efficient global modeling mechanisms. However, current literature predominantly evaluates these paradigms in isolation, under heterogeneous datasets and experimental protocols, which obscures paradigm-level insights into data efficiency, robustness, and deployment feasibility for plant disease recognition. Furthermore, the reliability of perception models is a prerequisite for effective downstream applications in smart farming. Recent evaluations of learning-based models for crop recommendation have underscored that errors in upstream disease classification can propagate through the decision-support pipeline, leading to suboptimal agricultural advice (Bakr et al., 2025). This research gap motivates us to conduct a unified and systematic comparative evaluation. It aims to clarify the practical advantages and limitations of different vision paradigms in realistic agricultural scenarios.

## 3 Data and methodology

### 3.1 Dataset description

The dataset used in this study was constructed by integrating images from two publicly available datasets on Kaggle: Plants Diseases Dataset and Balanced Cabbage Diseases Dataset (200 Each)^1^. By combining these sources, the resulting dataset covers 32 pest and disease categories across seven crop species, including bean, cabbage, chili, corn, potato, tea, and tomato. The 32 disease categories were mapped into six visual symptom groups (**Table 1**) based on established morphological definitions of plant disease symptoms described in classical plant pathology literature, complemented by modern studies in plant disease phenotyping (Agrios, 2005; Mahlein, 2016). Following the symptom typology, we categorized diseases exhibiting discrete lesions as ‘Localized Spots’, those with coalesced tissue death as ‘Diffuse Necrosis’, and surface fungal growths as ‘Surface Growth’, among others. This taxonomy ensures that our grouping aligns with biological reality while optimizing for visual feature extraction.

**Table 1:** Visual symptom-oriented classification framework.

Category no.	Disease and pest categories	Symptom type	Host plant species	Key visual characteristics
1	Anthracnose	Spot	Bean	Discrete localized spots exhibiting necrosis, concentric ring structures, and clear lesion margins
2	Rust		Bean	
3	Alternaria leaf spot		Cabbage	
4	Ring spot		Cabbage	
5	Bacterial leaf spot		Chili	
6	Cercospora leaf spot gray		Corn	
7	Common rust		Corn	
8	Red spot		Tea	
9	Bacterial spot		Tomato	
10	Septoria spot		Tomato	
11	Black rot	Necrosis	Cabbage	Coalesced lesions leading to extensive tissue death, often with irregular or diffuse margins
12	Northern leaf bight		Corn	
13	Early blight		Potato	
14	Late blight		Potato	
15	Brown blight		Tea	
16	Gray blight		Tea	
17	Early blight		Tomato	
18	Late blight		Tomato	
19	Downy mildew	Surface-colonizing	Cabbage	Powdery, fuzzy, or algal-like textures growing on the leaf surface, altering surface reflectance
20	Leaf mold		Tomato	
21	Algal		Tea	
22	Yellow leaf curl	Mosaic	Chili	Chlorotic mosaics, vein clearing, streaks, and structural deformities (curling) affecting the whole leaf or large sections
23	Mosaic virus		Tomato	
24	Yellow leaf curl		Tomato	
25	Aphid colony	Insect	Cabbage	Visible clustering of insect bodies and physical chewing damage (notches), distinct from pathogenic lesions.
26	Healthy	Healthy	Bean	Uniform green coloration with no visible lesions, discoloration, or deformities
27			Cabbage	
28			Chili	
29			Corn	
30			Potato	
31			Tea	
32			Tomato	

The integrated dataset contains 40,962 images, which were divided into a training set (38,352 images) and a test set (2,610 images). We used stratified sampling to partition the dataset, which helped preserve the original class ratio across all categories. Category labels were automatically extracted from file names according to the naming conventions of the source datasets. The images were standardized to a 384 × 384pixel spatial resolution, to ensure consistency during model training and evaluation.

To ensure a fair and controlled comparison across different model paradigms, the experiments were carried out under a unified experimental protocol. Specifically, all models were trained and evaluated on the same integrated dataset, using identical data splits, consistent preprocessing procedures, and the same evaluation metrics. This design eliminates confounding factors caused by dataset variation or experimental settings, enabling a reliable comparison of model performance and allowing performance differences to be attributed primarily to architectural characteristics.

Most images in the dataset depict individual leaves or localized regions rather than complete plant scenes captured in natural agricultural environments. As a result, backgrounds are relatively simple and disease symptoms are visually prominent. While this setting facilitates the analysis of symptom-level visual patterns, it differs from real-world field conditions, where images often involve complex backgrounds, occlusion, multiple plants, and varying illumination. Therefore, a potential domain gap may exist between this benchmark dataset and real-world deployment scenarios. Nevertheless, integrating multiple public datasets introduces variability in symptom appearance and imaging conditions, providing a more diverse and challenging benchmark for comparative evaluation.

To simulate realistic agricultural scenarios where annotated disease images are often limited, we designed multiple training regimes with progressively varying data availability (1%, 10%, 30%, and 100% of the full training set). The 100% setting corresponds to the complete training dataset and serves as a reference for upper-bound performance. The reduced subsets (1%, 10%, and 30%) were generated to represent different levels of data scarcity commonly encountered in practice: 1% corresponds to an extremely low-data regime, 10% represents a low-data scenario, and 30% approximates a moderately sufficient dataset. These thresholds were selected through iterative preliminary experiments to balance computational efficiency with meaningful performance evaluation across distinct data-scarcity regimes. All subsets were generated by random sampling while preserving the original class distribution. The test set (2,610 images) remained unchanged across all experiments to ensure fair comparison of model generalization performance.

### 3.2 Brief description of the used deep learning architectures

From the perspective of plant disease phenotyping, effective recognition models must jointly capture fine-grained lesion characteristics and long-range spatial context, while remaining robust to data scarcity and environmental noise. Plant pest and disease image classification therefore poses methodological challenges that differ fundamentally from generic object recognition tasks, as disease symptoms often appear as subtle lesion textures, irregular color variations, mildew patterns, and morphological distortions distributed heterogeneously across leaf surfaces (Salman et al., 2025). These challenges are further exacerbated under real agricultural conditions, where images are captured with cluttered backgrounds, variable illumination, and limited an-notated data, placing stringent demands on feature representation, contextual modeling, and generalization ability (Dhruw et al., 2025; Rodríguez-Lira et al., 2024).

**Figure 1:**
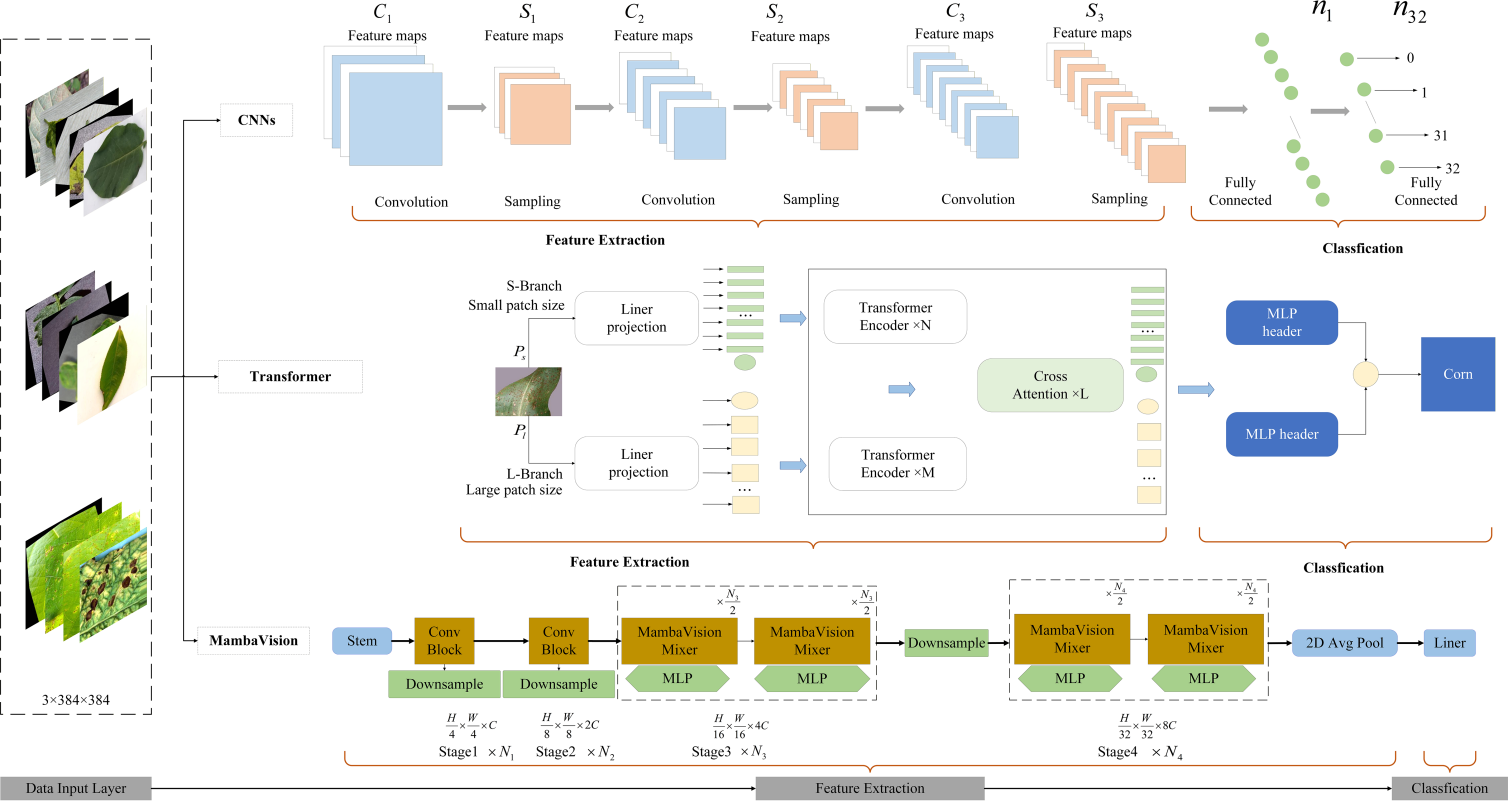
Technical workflow.

Different deep learning architectures address these challenges through distinct modeling mechanisms. CNN-based models primarily emphasize localized feature extraction, Transformer-based vision models focus on global dependency modeling via attention mechanisms, and hybrid MambaVision-based models aim to balance local representation and global context modeling with improved computational efficiency. To systematically investigate how these architectural paradigms influence plant pest and disease recognition performance, we benchmark representative CNN-, Transformer-, and hybrid SSM-based models under a unified experimental protocol, as illustrated in **Figure 1**.

#### 3.2.1 CNN-based models

CNNs constitute the foundational paradigm for image-based plant disease and pest recognition. By applying localized convolutional kernels across spatial neighborhoods, CNNs effectively capture low-level textures, color distributions, and edge structures, which are essential for identifying visually salient disease symptoms such as necrotic spots, mildew patterns, and leaf deformation (El Sakka et al., 2025).

However, CNNs inherently rely on limited receptive fields, and long-range contextual information can only be captured through deep stacking of convolutional layers. As a result, CNNs may struggle to distinguish disease categories that exhibit similar local textures but differ in their global spatial distribution or lesion organization (Ghuge et al., 2025). To explore how different CNN design strategies mitigate these limitations, a diverse set of CNN architectures is included in this benchmark. The selected CNN models can be broadly grouped into three architectural strategies:

(1) Classical deep convolutional architectures, represented by VGG and DenseNet.

VGG constructs deep networks by stacking small convolutional kernels, offering conceptual simplicity but incurring high computational and memory costs (Kumar et al., 2022; Zhao et al., 2023). DenseNet enhances feature reuse by connecting each layer to all subsequent layers, improving parameter efficiency and gradient flow, albeit with increased computational overhead (Zhang et al., 2023).

(2) Residual-enhanced architectures, including ResNet, ResNeXt, Wide Residual Networks (WRN), and V (ResNet_PReAct).

Residual learning enables stable optimization of deep networks by alleviating vanishing gradient is-sues (Xie et al., 2017). ResNeXt further introduces grouped convolutions to increase representational diversity, while WRN enhances model capacity by widening layers rather than increasing depth (Priya and Bharathi, 2025; Lak et al., 2024). ResNet_PReAct improves gradient propagation by reordering normalization and activation layers (Shafiq and Gu, 2022).

(3) Attention-augmented architectures, such as SE-ResNet-PReAct and Shake-Shake networks.

SE-ResNet-PReAct incorporates channel-wise attention to adaptively recalibrate feature responses, emphasizing informative channels relevant to disease patterns. Shake-Shake networks introduce stochastic regularization by randomly mixing parallel branches during training, which can improve generalization on limited datasets (Zhao et al., 2024; Yamada et al., 2019).

Together, these models enable systematic analysis of how local feature learning, residual optimization, attention mechanisms, and regularization influence classification performance.

#### 3.2.2 Transformer-based vision models

Transformer-based vision models address the locality constraint of CNNs by employing self-attention mechanisms that explicitly model long-range dependencies across the entire image. By treating images as sequences of tokens, Transformers enable global contextual reasoning, which is particularly advantageous for recognizing diseases characterized by spatially dispersed or multi-region symptoms (Khan et al., 2023; Han et al., 2022).

Despite these advantages, Transformer models face notable methodological challenges in agricultural applications. The quadratic computational complexity of self-attention with respect to spatial resolution leads to substantial computational overhead for high-resolution images (Ji and Zhao, 2024). Moreover, Transformers often require large-scale datasets and strong regularization strategies to achieve stable convergence, which may limit their effectiveness in data-constrained agricultural scenarios (Guo et al., 2024; Liu et al., 2024a).

To capture a broad spectrum of Transformer design, this study evaluates the following representative models: DeiT, CrossViT, Focal Transformer, PVT-Small, and Twins, which would emphasize different strategies for balancing global attention and local feature modeling:

1. DeiT focuses on data-efficient training through distillation and regularization mechanisms, making it suitable for moderately sized datasets (Sun et al., 2025).
2. CrossViT introduces cross-scale token interactions, enabling simultaneous modeling of finegrained lesion textures and global leaf structures (Siddiqui et al., 2025).
3. Focal Transformer enhances local attention while progressively expanding the receptive field, facilitating recognition of small and spatially scattered disease symptoms (Lin et al., 2024; Shi et al., 2025).
4. PVT-Small and Twins adopt hierarchical and pyramid-based designs that bridge CNN-style multiscale representations with Transformer-based global modeling, improving efficiency and scalability (Liu et al., 2024b, Liu et al., 2023).

By evaluating these diverse architectures, the study investigates how different attention mechanisms and hierarchical designs influence feature representation and classification performance in pest and disease recognition tasks.

#### 3.2.3 Hybrid MambaVision-based models

MambaVision represents a hybrid modeling paradigm that integrates convolutional feature extraction with selective SSMs and Transformer-style components. Unlike conventional Transformers that rely on self-attention, MambaVision employs SSM-based sequence modeling to capture long-range dependencies with linear computational complexity, offering improved efficiency for high-resolution visual inputs (Hatamizadeh and Kautz, 2025; Liu et al., 2025b).

In MambaVision, convolutional layers are responsible for extracting local texture and structural features, preserving sensitivity to fine-grained disease patterns. Mamba modules then model global context through state-space dynamics, enabling efficient long-range dependency modeling without the quadratic cost of attention mechanisms. Transformer components are further employed for high-level semantic integration (Qu et al., 2024). For image data, the Mamba module is adapted by replacing one-dimensional causal convolutions with two-dimensional convolutions and introducing symmetric parallel branches to preserve spatial coherence (Rahman et al., 2024; Xu et al., 2024). This design allows the model to maintain local feature fidelity while benefiting from global contextual modeling through recurrent state updates. Consequently, hybrid MambaVision-based model forms a complementary architecture that addresses the limitations of purely convolutional or purely attention-based models, by combining CNN-based locality, SSM-based global modeling, and Transform-er-based semantic integration.

Multiple MambaVision variants are evaluated, including MambaVision-T, MambaVision-T2, Mam-baVision-S, MambaVision-B, MambaVision-L, and MambaVision-L2, along with their corresponding pre-trained (e.g., MambaVision -B-21K, MambaVision -L-21K) and high-resolution (e.g., MambaVision -L2-512-21K) configurations. These variants differ in network depth, channel width, and input resolution, allowing systematic analysis of the effects of model scale, pre-training, and spatial resolution on classification performance (Hatamizadeh and Kautz, 2025).

The 21K designation denotes pre-training on the ImageNet-21K dataset rather than the number of training epochs. By comparing pre-trained and non-pre-trained variants, the study evaluates the transferability of generic visual representations to fine-grained agricultural disease recognition. High-resolution variants with 512×512 input size are included to assess whether increased spatial detail benefits the identification of small lesions and subtle disease symptoms (Hatamizadeh and Kautz, 2025).

Through this diverse set of configurations, hybrid MambaVision-based models facilitate comprehensive investigation of the roles of architectural scale, pre-training, and input resolution in agricultural image classification tasks.

## 4 Results and analysis

### 4.1 Evaluation metrics

To comprehensively evaluate the pest and disease recognition performance of different deep learning architectures, we employed accuracy, precision, recall, and F1-score as quantitative metrics, defined by Equations 1-4.

(1) $$accuracy=\frac{TP+TN}{TP+FP+FN+TN}$$

(2) $$precision=\frac{TP}{TP+FP}$$

(3) $$recall=\frac{TP}{TP+FN}$$

(4) $$F1-score=\frac{2\times precision\times recall\;}{precision+recall\;}$$

where TP denotes True Positives (correctly identified positive instances), FP represents False Positives (incorrectly identified negative instances), FN signifies False Negatives (missed positive instances), and TN indicates True Negatives (correctly identified negative instances).

### 4.2 Implementation details

All experiments were implemented in Python 3.10 using PyTorch 2.1.0 with CUDA 12.1 on an Ubuntu 22.04 workstation equipped with an Intel Xeon E5–2680 v4 CPU and an NVIDIA GeForce RTX 3060 GPU (12 GB VRAM). For reproducibility, random seeds were fixed for Python/NumPy/PyTorch (including CUDA) and deterministic CuDNN behavior was enabled. Unless stated, the models were trained with the Adam optimizer ( ${\text{β}}_{1}=0.9$, ${\text{β}}_{2}=0.999$, weight decay=0.0) using a cosine learning-rate decay schedule. Furthermore, we trained the model for 120 epochs with a batch size of 8 and an initial learning rate of $10^{-4}$, with the minimum learning rate set to 1% of the initial value.

To ensure a fair and controlled comparison across different model architectures, all models were trained and evaluated under a unified experimental protocol. Specifically, identical data splits, preprocessing procedures, augmentation strategies, optimization settings, training epochs, and evaluation metrics were applied across all models. Classification heads were consistently adapted to the same set of pest and disease categories. Multiple independent runs were conducted to account for training stochasticity, and consistent performance trends were observed across runs. This standardized setup ensures that performance differences can be attributed primarily to architectural characteristics rather than variations in training configuration.

### 4.3 Training divergence dynamics with the increasing number of training samples

**Figure 2:**
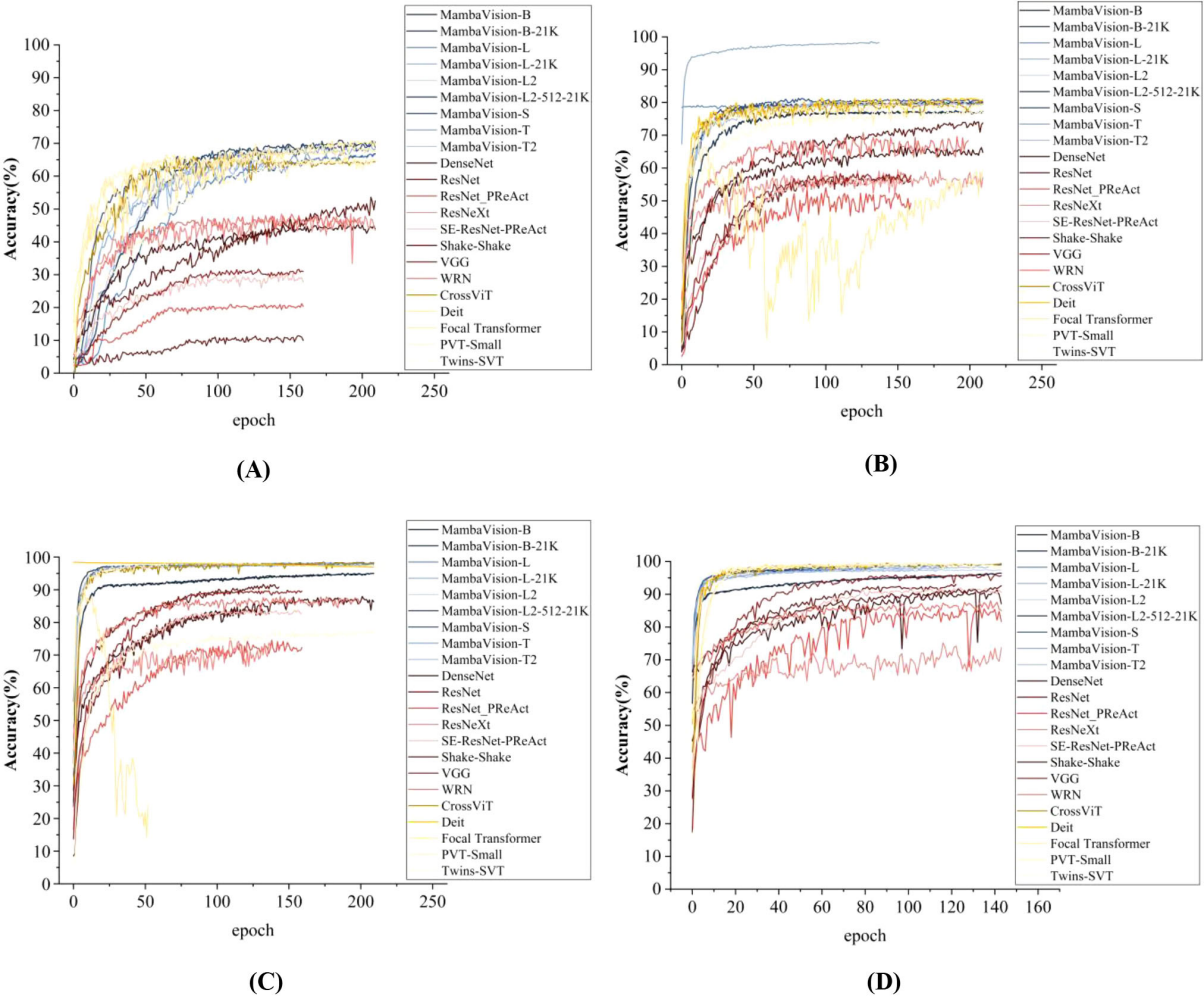
Fluctuation of Training Accuracy for Different Deep Learning Architectures as the Number of Training Samples Rises. **(A)** Training Accuracy with Set Size: 1%; **(B)** Training Accuracy with Set Size: 10%; **(C)** Training Accuracy with Set Size: 30%; **(D)** Training Accuracy with Set Size: 100%. **Figure 2** illustrates the training accuracy dynamics of different deep learning architectures across training epochs for the 32-class plant pest and disease classification task, under four distinct training set sizes (1%, 10%, 30%, and 100%).

Dataset size plays a critical role in deep learning model training and generalization. **Figure 2** illustrates the training accuracy dynamics of different architectures across epochs for the 32-class plant pest and disease classification task, under four training set sizes (1%, 10%, 30%, and 100%), enabling a systematic evaluation from extremely low- to full-data regimes, as well as an implicit analysis of training error and prediction stability.

Across all architectures, three consistent and robust trends occur with the training set size increasing. First, final classification accuracy increased monotonically with data availability. For instance, MambaVision-L2-512-21K achieved approximately 70% accuracy when trained with only 1% of the data (**Figure 2A**), which gradually improved to nearly 100% under the full dataset setting (**Figure 2D**). Second, convergence speed improved substantially: when trained with 100% samples, most models reached 80% accuracy within 20 epochs, whereas more than 50 epochs were required under the 1% training regime. Third, training stability increased with dataset size, as accuracy fluctuations decreased from over 20% with the smallest dataset to below 5% with the full dataset, resulting in notably smoother learning curves. From an error analysis perspective, these reduced fluctuations directly reflect lower training variance and more stable gradient updates, indicating better control over overfitting and prediction error in data-rich regimes. Similar trends were observed on the held-out test set, indicating that larger datasets improve both learning efficiency and generalization robustness.

Clear performance stratification among architectures was observed consistently across all training set sizes. Hybrid MambaVision-based models formed the top-performing group, achieving 60–70% accuracy even with only 1% training samples and approaching saturation under full-data conditions (**Figure 2**). This robustness is consistent with their local–global feature modeling capability, which facilitates the joint representation of fine-grained lesion patterns and global leaf morphology. In terms of error behavior, MambaVision models exhibit the smoothest learning curves with minimal oscillations, indicating lower prediction variance and more stable error propagation during training. In contrast, CNN-based models (e.g., ResNet) exhibited limited small-sample generalization and required at least 30% of the training data to exceed 70% accuracy (**Figure 2C**). Their learning curves show larger fluctuations and slower convergence, reflecting higher training error and greater sensitivity to noisy gradient updates in low-data regimes. Different from Hybrid MambaVision-based and CNN-based models, Transformer-based models (e.g., DeiT) achieved competitive performance under larger datasets but showed pronounced instability—up to 15% accuracy fluctuation—when trained with 10% samples or fewer (**Figure 2B**), suggesting increased sensitivity to data scarcity and elevated prediction uncertainty. It should be noted that lightweight architectures such as PVT-Small consistently underperformed in low-data regimes, achieving below 20% accuracy with 1% samples (**Figure 2A**), indicative of insufficient representational capacity to mitigate overfitting and control prediction error for this task.

Overall, these results are consistent with the fine-grained characteristics of 32-class pest and disease recognition, where subtle lesion textures and localized visual cues must be interpreted within broader leaf-level context. Architectures capable of efficient long-range dependency modeling demonstrate superior robustness under limited data conditions, as evidenced by their stable learning curves and controlled error propagation, whereas models relying primarily on local convolutional features or reduced parameterization show constrained performance and stability, with higher prediction variance and more pronounced training error under data scarcity.

### 4.4 Classification performance from quantitative perspectives

**Figure 3:**
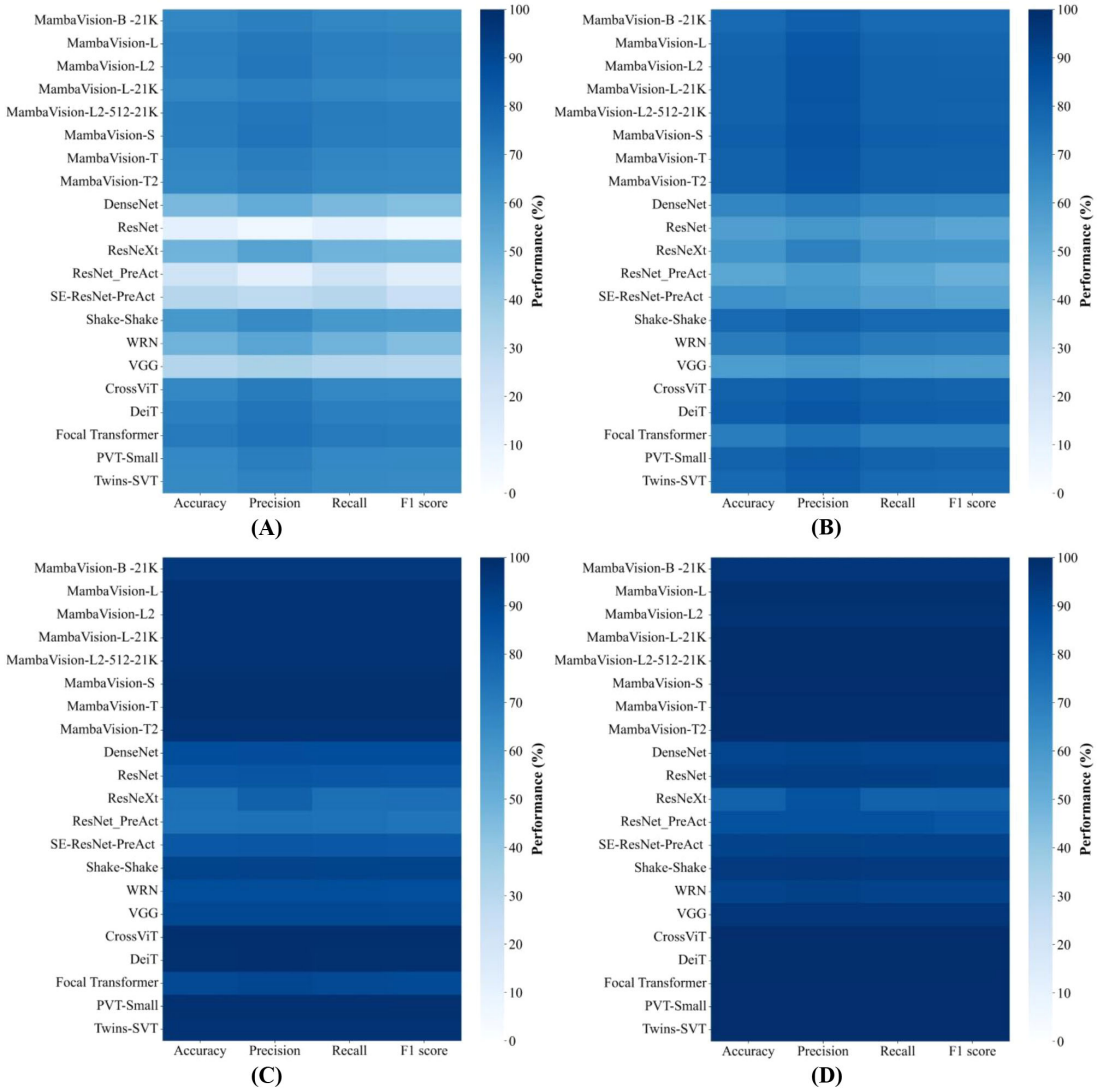
Model’s Performance under Varying Training Set Sizes. **(A)** Heatmap of model performance (Training set size = 1%); **(B)** Heatmap of model performance (Training set size = 10%); **(C)** Heatmap of model performance (Training set size = 30%); **(D)** Heatmap of model performance (Training set size = 100%). This Figure presents heatmaps summarizing the classification performance of 22 deep learning architectures on the 32-class plant pest and disease classification task. The heatmap color gradient represents performance values (0–100%), with four key metrics (accuracy, precision, recall, F1-score) displayed across columns.

**Figure 3** summarizes the classification performance of different models under varying training set sizes, revealing a strong dependence of recognition accuracy on data availability. To complement the visual comparison, **Tables 2**–**5** report detailed quantitative metrics, including accuracy, precision, recall, and F1-score, under four representative training regimes (1%, 10%, 30%, and 100% of the training set), providing numerical support for the observed trends.

**Table 2:** Comparison of multi-dimensional performance metrics of various networks under training set size 1%.

Model category	Network architecture	Accuracy (%)	Precision (%)	Recall (%)	F1-score (%)
Hybrid MambaVision-based models	MambaVision-B	66.78	69.59	66.78	65.84
	MambaVision-B -21K	66.90	69.63	66.90	65.90
	MambaVision-L	69.92	72.07	69.92	69.00
	MambaVision-L2	69.39	72.28	69.39	68.65
	MambaVision-L-21K	67.39	69.74	67.39	66.29
	MambaVision-L2-512-21K	71.00	72.45	71.00	70.28
	MambaVision-S	70.69	73.46	70.69	70.20
	MambaVision-T	67.24	70.56	67.24	66.63
	MambaVision-T2	66.74	69.03	66.74	66.24
CNN-based models	DenseNet	46.36	51.87	46.36	43.94
	ResNet	11.42	6.68	11.42	7.15
	ResNeXt	48.74	55.61	48.74	47.76
	ResNet_PReAct	21.15	12.37	21.15	13.44
	SE-ResNet-PReAct	30.57	28.42	30.57	24.66
	Shake-Shake	60.46	65.16	60.46	59.31
	WRN	48.47	54.97	48.47	44.40
	VGG	31.61	34.44	31.61	30.32
Transformer-based models	CrossViT	66.67	70.75	66.67	66.30
	DeiT	69.89	73.22	69.89	69.42
	Focal Transformer	71.53	73.92	71.53	70.89
	PVT-Small	66.13	70.09	66.13	65.95
	Twins-SVT	65.59	68.06	65.59	64.91

**Table 3:** Comparison of multi-dimensional performance metrics of various networks under training set size 10%.

Model category	Network architecture	Accuracy (%)	Precision (%)	Recall (%)	F1-score (%)
Hybrid MambaVision-based models	MambaVision-B	77.47	80.92	77.47	77.06
	MambaVision-B -21K	77.55	81.15	77.55	77.12
	MambaVision-L	79.35	83.62	79.35	79.18
	MambaVision-L2	80.19	84.42	80.19	80.02
	MambaVision-L-21K	80.31	84.50	80.31	80.10
	MambaVision-L2-512-21K	80.27	84.08	80.27	79.98
	MambaVision-S	81.26	84.85	81.26	80.93
	MambaVision-T	80.42	84.19	80.42	80.14
	MambaVision-T2	80.27	83.92	80.27	80.00
CNN-based models	DenseNet	67.20	70.98	67.20	66.42
	ResNet	57.47	60.88	57.47	54.97
	ResNeXt	61.65	69.10	61.65	61.25
	ResNet_PReAct	54.02	58.93	54.02	50.16
	SE-ResNet-PReAct	62.51	60.70	57.05	55.26
	Shake-Shake	77.39	80.11	77.39	77.00
	WRN	70.80	74.28	70.80	70.08
	VGG	58.31	60.65	58.31	57.80
Transformer-based models	CrossViT	80.34	82.11	80.34	79.50
	DeiT	81.49	84.36	81.49	81.00
	Focal Transformer	70.69	75.02	70.69	70.34
	PVT-Small	79.92	83.05	79.92	79.27
	Twins-SVT	77.93	81.86	77.93	77.52

**Table 4:** Comparison of multi-dimensional performance metrics of various networks under training set size 30%.

Model category	Network architecture	Accuracy (%)	Precision (%)	Recall (%)	F1-score (%)
Hybrid MambaVision-based models	MambaVision-B	94.98	95.02	94.98	94.96
	MambaVision-B -21K	95.06	95.11	95.06	95.03
	MambaVision-L	97.51	97.54	97.51	97.51
	MambaVision-L2	97.59	97.62	97.59	97.58
	MambaVision-L-21K	97.43	97.49	97.43	97.43
	MambaVision-L2-512-21K	97.66	97.70	97.66	97.65
	MambaVision-S	98.16	98.18	98.16	98.16
	MambaVision-T	98.39	98.41	98.39	98.40
	MambaVision-T2	97.74	97.76	97.74	97.74
CNN-based models	DenseNet	88.12	88.52	88.12	87.94
	ResNet	84.18	84.43	84.18	83.60
	ResNeXt	75.25	80.67	75.25	75.47
	ResNet_PReAct	74.56	74.91	74.56	73.48
	SE-ResNet-PReAct	83.83	84.08	83.83	83.39
	Shake-Shake	91.69	92.07	91.69	91.57
	WRN	88.08	88.65	88.08	87.82
	VGG	89.89	90.01	89.89	89.78
Transformer-based models	CrossViT	98.54	98.56	98.54	98.54
	DeiT	98.43	98.46	98.43	98.43
	Focal Transformer	89.62	90.63	89.62	89.45
	PVT-Small	98.31	98.33	98.31	98.31
	Twins-SVT	97.78	97.80	97.78	97.77

**Table 5:** Comparison of multi-dimensional performance metrics of various networks under training set size 100%.

Model category	Network architecture	Accuracy (%)	Precision (%)	Recall (%)	F1-score (%)
Hybrid MambaVision-based models	MambaVision-B	96.44	96.46	96.44	96.41
	MambaVision-B -21K	96.25	96.28	96.25	96.23
	MambaVision-L	98.24	98.26	98.24	98.22
	MambaVision-L2	97.78	97.81	97.78	97.76
	MambaVision-L-21K	98.89	98.90	98.89	98.89
	MambaVision-L2-512-21K	99.16	99.17	99.16	99.16
	MambaVision-S	99.27	99.28	99.27	99.27
	MambaVision-T	98.62	98.63	98.62	98.62
	MambaVision-T2	98.54	98.58	98.54	98.54
CNN-based models	DenseNet	91.38	91.74	91.38	91.12
	ResNet	92.99	93.24	92.99	92.71
	ResNeXt	80.21	85.67	80.21	80.47
	ResNet_PReAct	85.94	86.50	85.94	84.53
	SE-ResNet-PReAct	92.15	92.56	92.15	91.95
	Shake-Shake	94.69	95.07	94.69	94.57
	WRN	92.13	92.65	92.13	91.82
	VGG	96.21	96.29	96.21	96.17
Transformer-based models	CrossViT	99.54	99.55	99.54	99.54
	DeiT	99.16	99.18	99.16	99.16
	Focal Transformer	99.50	99.51	99.50	99.51
	PVT-Small	99.43	99.43	99.43	99.42
	Twins-SVT	99.54	99.55	99.54	99.54

#### 4.4.1 Performance under extreme data scarcity (1%)

When trained with extremely limited data (1% training set; **Figure 3A**), all models experience substantial performance degradation, indicating insufficient feature learning and severely constrained generalization capability. Classical CNN architectures are the most affected in this regime, with accuracies generally below 30%. As shown in **Table 2**, ResNet achieves only 11.42% accuracy and 6.68% precision, VGG reaches 31.61% accuracy, and DenseNet attains 46.36% accuracy, reflecting their strong reliance on large-scale labeled data.

In contrast, Transformer-based models exhibit improved robustness under data scarcity, achieving moderate performance levels. Representative models such as DeiT and CrossViT reach 69.89% and 66.67% accuracy, respectively, at the 1% training set. Notably, the MambaVision family consistently outperforms all other architectures in this low-resource regime, achieving accuracies in the range of approximately 60-80%. The best-performing variant reaches 71.00% accuracy, highlighting superior data efficiency under extreme sample scarcity.

#### 4.4.2 Performance evolution with increasing data availability

As the training set size increases, performance improves monotonically across all architectures and evaluation metrics. The most pronounced gains occur when the training data expand from 1% to 10% (**Figures 3A, B**), whereas further increases yield progressively smaller improvements, indicating diminishing marginal returns from additional data. This trend is consistently observed across model families. For ex-ample, the accuracy of a representative MambaVision variant increases sharply from 66.78% at 1% training data to 77.47% at 10%, followed by a larger improvement to 94.98% at 30%, and finally a marginal gain to 96.44% when trained on the full dataset.

#### 4.4.3 Performance under data-sufficient regimes (30% and 100%)

Under moderate and large-scale training conditions (30% and 100%), MambaVision models approach near-saturated performance, achieving approximately 90-100% across all evaluation metrics. As shown in **Table 4**, the differences among MambaVision variants become negligible at full data scale, with accuracy, precision, recall, and F1-score differing by less than 0.1 percentage points. This convergence indicates strong generalization capability and well-balanced predictive behavior once sufficient training data are available.

Across all data regimes, a clear performance stratification among architectural families is consistently observed. Transformer-based models generally outperform CNN baselines by leveraging global self-attention mechanisms, particularly under moderate and large data availability. However, their performance remains slightly inferior to that of MambaVision. For instance, although DeiT achieves competitive accuracy at both low and moderate data scales, it does not surpass MambaVision under either data-limited or data-sufficient conditions. This gap may be attributed to the quadratic computational complexity of self-attention, which limits efficiency in modeling long visual sequences. In contrast, the selective scanning mechanism employed by Mamba architectures enables more scalable and effective modeling of long-range dependencies.

#### 4.4.4 Robustness analysis via continuous performance curves

**Figure 4:**
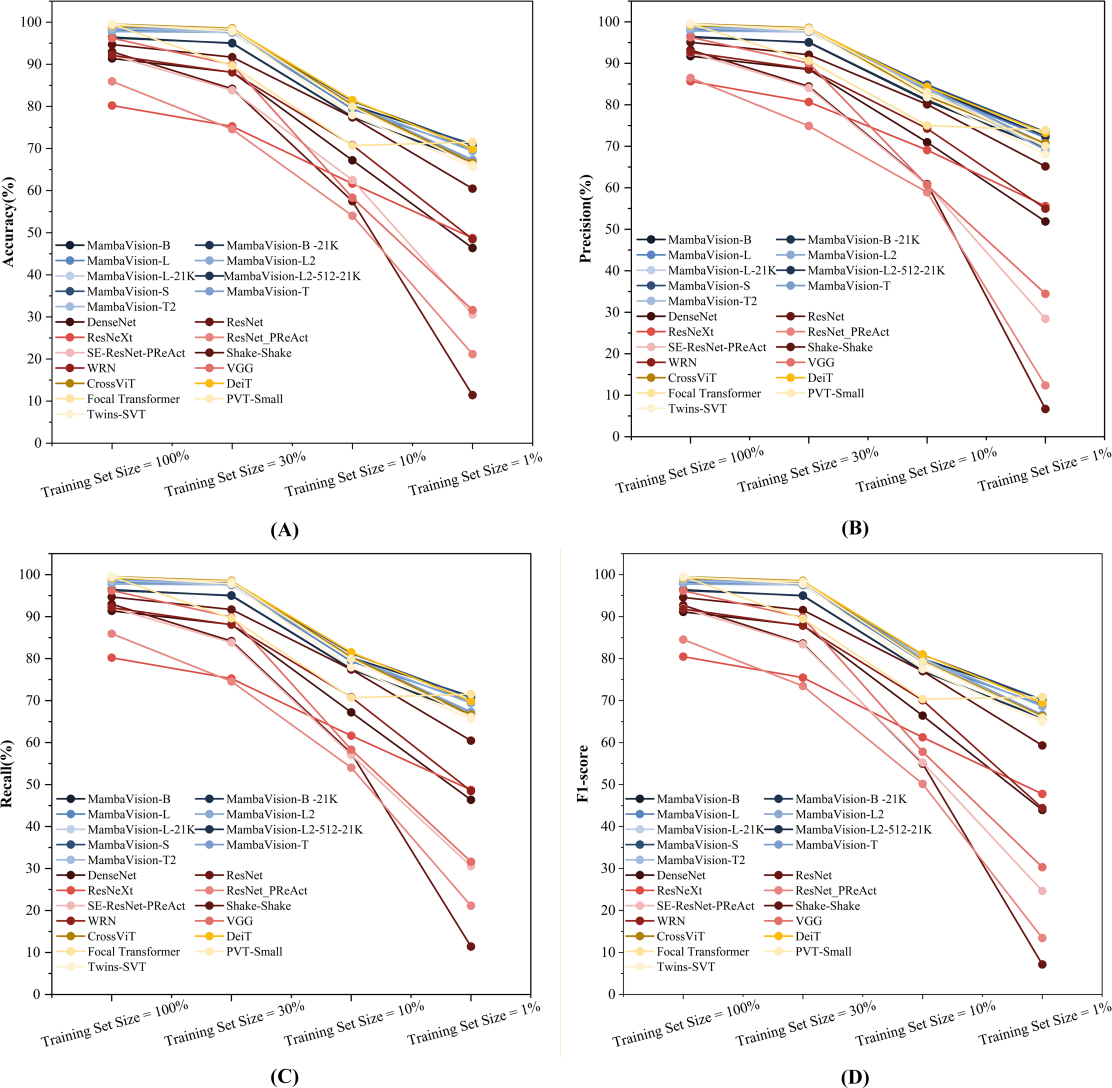
Comprehensive performance degradation analysis across different architectures under progressively reduced training data (100% → 1%). **(A)** Accuracy Curves; **(B)** Precision Curves; **(C)** Recall Curves; **(D)** F1-score Curve.

To further examine performance robustness, complementary line plot analyses were conducted to visualize the evolution of model performance across training set sizes for all four evaluations metrics, as shown in **Figure 4**. These curves directly capture the rate of performance degradation as the training data are reduced from 100% to 1%. MambaVision models exhibit a gradual and relatively mild decline, whereas classical CNNs experience a sharp drop, providing direct evidence of superior robustness under low-resource conditions.

This behavior is quantitatively supported by the retention ratio of accuracy at the 1% training set relative to full data (**Figure 4A**). A representative MambaVision variant retains approximately 69% of its full-data accuracy when trained with only 1% of the data, whereas ResNet and VGG retain only about 12% and 33%, respectively. In addition, the performance curves of MambaVision models remain closely aligned across accuracy, precision, recall, and F1-score, indicating balanced predictive behavior without evident trade-offs among metrics (**Figures 4A-D**).

In contrast, classical CNNs exhibit pronounced precision–recall imbalance under extreme data scarcity (**Figures 4B, C**). For example, ResNet achieves a recall of 11.42% but only 6.68% precision at the 1% training set (**Table 2**), reflecting unstable and poorly calibrated predictions. Furthermore, the line plots reveal consistent advantages from both pretraining and increased model capacity within the MambaVision family. Pretrained variants out-perform their non-pretrained counterparts in low-data regimes, and larger-capacity models achieve higher accuracy across all training set sizes, confirming that both factors contribute to improved robustness under data-limited conditions.

With regards to F1-score (**Figure 4D**), which synthesizes precision and recalls into a single measure of overall predictive quality, the trend is consistent with other metrics: MambaVision models retain a significantly higher proportion of their full-data F1-score at 1% training data compared to all classical CNNs. Classical models like ResNet and VGG see their F1-score drop far more steeply, confirming that the precision–recall imbalance directly translates to severely degraded overall performance in data-limited scenarios.

#### 4.4.5 Symptom-level comparison of model paradigms under 30% training regimes

**Table 6** summarizes symptom-level comparison in F1-score of model paradigms under training set size 30%. The symptom-wise comparison reveals that performance differences among model paradigms are closely linked to symptom morphology and spatial organization. CNN-based models perform competitively on spot-type symptoms dominated by localized texture cues, while Transformer-based models show stronger global modeling capacity but higher variability under limited training data. In contrast, MambaVision consistently achieves high and stable F1-scores across symptom groups that require joint modeling of fine-grained textures and long-range spatial distribution, highlighting its suitability for complex plant disease patterns characterized by both local and global visual dependencies.

**Table 6:** Symptom-level comparison in F1-score of model paradigms under training set size 30%.

Model category	Network architecture	Symptom type					
		Spot	Necrosis	Surface-colonizing	Mosaic	Insect	Healthy
Hybrid MambaVision-based models	MambaVision-B	97.12	96.51	95.67	98.45	99.17	96.48
	MambaVision-B -21K	97.17	96.45	94.74	97.95	99.17	96.66
	MambaVision-L	98.33	98.10	96.77	99.74	99.17	98.25
	MambaVision-L2	98.40	98.28	97.24	99.74	99.17	98.75
	MambaVision-L-21K	98.40	97.89	97.00	99.74	99.17	98.34
	MambaVision-L2-512-21K	98.21	98.95	96.30	99.22	100.00	98.75
	MambaVision-S	98.65	98.81	98.39	99.48	98.36	98.83
	MambaVision-T	99.04	98.75	97.03	99.74	99.16	99.58
	MambaVision-T2	98.72	98.55	97.24	99.74	99.17	98.66
CNN-based models	DenseNet	91.36	90.79	83.41	93.88	79.37	91.39
	ResNet	87.15	89.85	80.49	95.36	63.87	90.72
	ResNeXt	84.84	83.31	69.13	75.71	82.61	81.44
	ResNet_PReAct	80.03	80.15	58.73	87.92	38.38	84.82
	SE-ResNet-PReAct	87.16	87.31	79.81	92.82	59.62	91.18
	Shake-Shake	95.21	92.68	88.18	98.44	88.06	93.72
	WRN	92.74	90.19	75.42	95.90	85.50	92.05
	VGG	92.51	92.33	88.13	96.88	90.48	94.02
Transformer-based models	CrossViT	96.99	97.42	97.18	95.59	99.17	97.35
	DeiT	96.90	96.31	93.84	94.96	95.93	96.33
	Focal Transformer	90.56	91.39	84.43	96.30	76.06	92.61
	PVT-Small	97.41	95.79	92.59	98.51	95.80	97.34
	Twins-SVT	97.38	98.17	92.73	98.55	93.91	98.09

### 4.5 Computational efficiency comparison

To quantitatively assess computational efficiency and deployment feasibility, **Table 7** compares Hybrid MambaVision-based, CNN-based, and Transformer-based models in terms of FLOPs, inference speed (FPS), GPU memory consumption, and parameter count. Rather than focusing on individual metrics, this analysis emphasizes overall efficiency profiles and deployment trade-offs across architectural paradigms, providing practical guidance for model selection under diverse computational constraints.

**Table 7:** Efficiency comparison of different deep learning architectures in terms of computational complexity, inference speed, memory usage, and model parameters.

Model category	Network architecture	Flops (GFlops)	Inference speed (FPS)	GPU memory (MB)	Parameters (M)
Hybrid MambaVision-based models	MambaVision-B	110.244	128.31	1228.57	96.693
	MambaVision-B -21K	110.244	128.26	1228.57	96.693
	MambaVision-L	257.153	58.15	2861.81	226.442
	MambaVision-L2	281.108	53.19	2967.16	240.036
	MambaVision-L-21K	257.153	58.22	2861.81	226.442
	MambaVision-L2-512-21K	196.148	56.02	3050.35	240.036
	MambaVision-S	53.455	187.1	677.3	49.396
	MambaVision-T	33.419	209.34	414.28	31.174
	MambaVision-T2	39.245	171.69	440.74	34.484
CNN-based models	DenseNet	14.947	125.18	121.2	0.777
	ResNet	12.612	95.55	30.86	1.732
	ResNeXt	265.658	43.53	684.35	34.449
	ResNet_PReAct	12.61	132.44	27.83	1.732
	SE-ResNet-PReAct	12.711	56.45	28.09	1.75
	Shake-Shake	185.644	69.9	225.17	26.201
	WRN	257.376	70.14	305.74	36.493
	VGG	20.116	656.34	118.1	15.772
Transformer-based models	CrossViT	5.08	108.02	505.91	26.7
	DeiT	33.73	295.58	346	86.57
	Focal Transformer	8.83	44.08	511	27.52
	PVT-Small	9.62	62.51	389	23.6
	Twins-SVT	1.39	88.71	28	3.42

The three architectural families exhibit clearly differentiated efficiency regimes. Hybrid MambaVision-based models demonstrate a structured and predictable efficiency–capacity scaling behavior. Lightweight variants (T, T2, and S) achieve a favorable balance between computational cost and inference throughput, delivering high FPS with moderate FLOPs and memory usage, whereas larger variants (B, L, and L2) progressively trade efficiency for increased representational capacity. This monotonic scaling indicates that MambaVision supports controllable, deployment-oriented trade-offs, enabling flexible capacity adjustment without abrupt efficiency degradation. Notably, pre-trained and non-pre-trained variants share identical efficiency metrics, confirming that pretraining improves performance without additional computational cost.

In contrast, CNN-based models exhibit pronounced heterogeneity in efficiency characteristics. Some architectures (e.g., ResNet, ResNet_PReAct, and DenseNet) are highly lightweight in terms of FLOPs, parameters, and memory usage but provide only moderate FPS. Others display the opposite tendency: VGG achieves very high FPS at the expense of increased memory and parameter usage, while heavier architectures such as ResNeXt, WRN, and Shake-Shake incur substantial computational overhead with relatively low FPS. This wide dispersion suggests that CNNs often optimize isolated efficiency dimensions but lack balanced performance across metrics.

Transformer-based models generally emphasize lightweight computation, with several variants achieving low FLOPs, parameter counts, and memory consumption. Twins-SVT represents the most com-pact configuration, whereas CrossViT, Focal Transformer, and PVT-Small maintain low computational overhead with varying FPS. DeiT combines relatively higher parameter counts with competitive FPS, reflecting architectural optimizations for efficient inference. However, compared with MambaVision, Transformer-based models show less consistent inference behavior across architectures, indicating greater variability in deployment efficiency.

Overall, **Table 7** reveals distinct efficiency profiles among the three paradigms: MambaVision models provide scalable and well-balanced efficiency–performance trade-offs, CNN-based models exhibit extreme and heterogeneous behaviors, and Transformer-based models favor lightweight computation with variable inference stability. These differences underscore the practical advantages of hybrid state-space–based architectures for deployment-sensitive agricultural vision applications.

### 4.6 Trade-off between classification performance and computational efficiency

**Figure 5:**
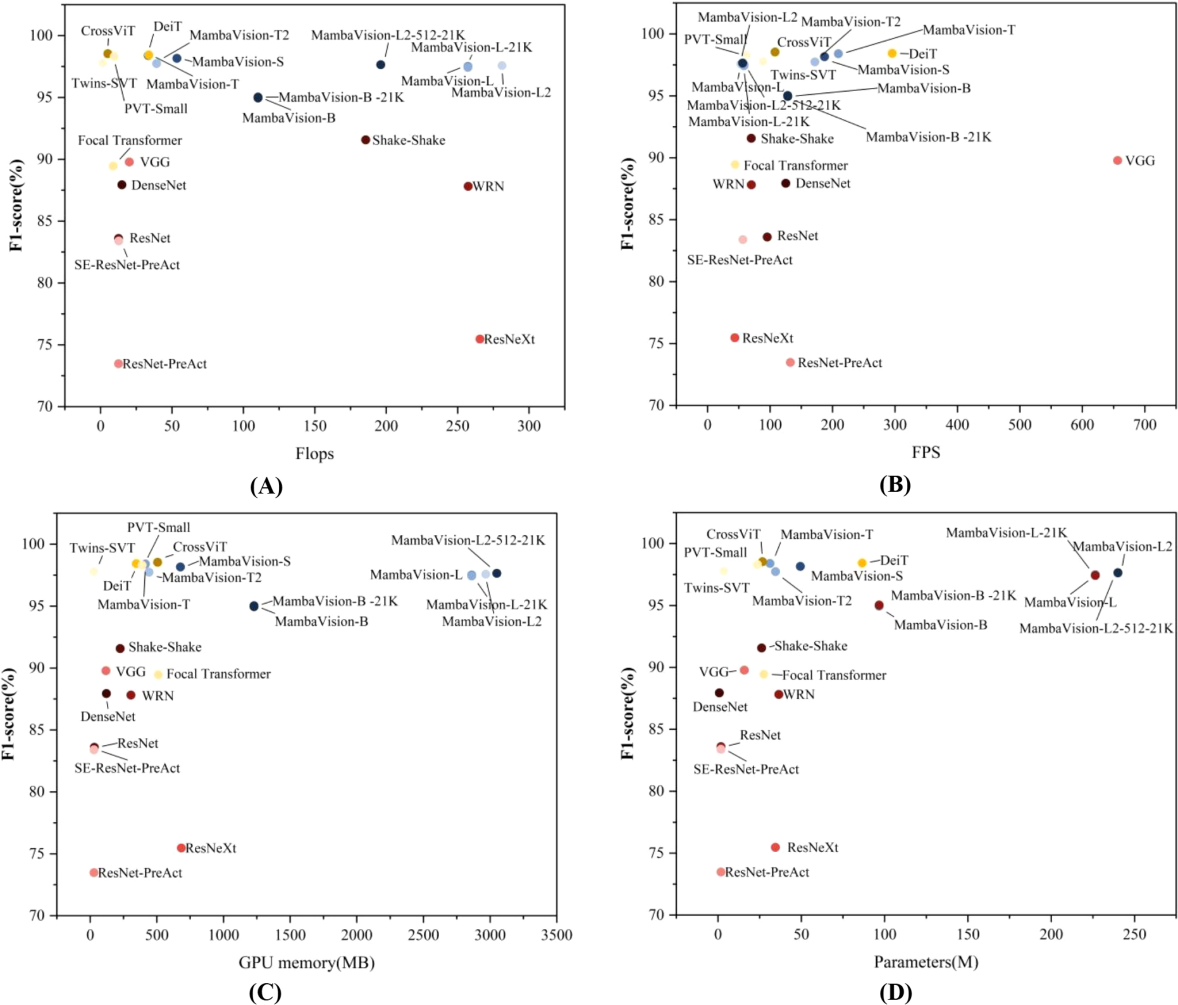
Trade-off between F1-Score and Computational Efficiency (Training Set Size = 30%). **(A)** F1-Score vs. Flops; **(B)** F1-Score vs. FPS; **(C)** F1-Score vs. GPU memory (MB); **(D)** F1-Score vs. Parameters (M).

To systematically characterize the trade-off between classification performance and computational efficiency, we analyze all trained models under a moderate data regime (training set size=30%), where both performance differentiation and efficiency constraints become clearly observable. **Figure 5** summarizes this analysis through scatter plots relating F1-score to Flops, FPS, GPU memory consumption, and parameter count, enabling a holistic view of the performance-efficiency landscape across architectural paradigms.

Across all four dimensions, a clear and consistent stratification emerges among model families, revealing three distinct operating regimes. CNN-based models concentrate in a low-cost, low-performance region; Transformer-based models occupy an intermediate zone characterized by strong efficiency but a limited performance ceiling; and MambaVision models dominate the high-performance regime while maintaining moderate and controllable computational overhead. This layered distribution reflects funda-mental architectural trade-offs and provides a principled basis for model selection under different deployment constraints.

From a computation-performance perspective (**Figure 5A**), MambaVision models convert increased Flops into substantial and consistent F1-score gains, occupying the high-F1 (95-100%) region with medium to high computational cost. In contrast, Transformer-based models achieve relatively competitive performance with significantly lower Flops, but their F1-scores plateau below those of MambaVision, indicating diminishing returns in accuracy despite computational efficiency. CNN-based models remain confined to a low-Flops, low-F1 region, suggesting that reduced computation alone cannot compensate for limited representational capacity.

In terms of inference efficiency (**Figure 5B**), MambaVision achieves a balanced compromise between accuracy and throughput. Within the family, a clear capacity-speed trade-off is observed: lightweight variants favor higher FPS, while larger variants prioritize peak performance. Transformer and CNN architectures can achieve very high FPS, but this advantage is systematically accompanied by reduced classification accuracy, highlighting a design bias toward throughput rather than expressive modeling. Notably, MambaVision remains competitive in FPS while consistently operating at a higher accuracy level, indicating superior efficiency-accuracy alignment.

The memory-performance relationship (**Figure 5C**) further reinforces this pattern. MambaVision models exhibit a gradual increase in GPU memory usage with model scale, yet this increase is proportional to substantial performance gains. Transformer-based models demonstrate strong memory efficiency but fall short of peak accuracy, while CNN-based models are extremely memory-light but constrained by a pronounced performance ceiling. This positions MambaVision as a middle ground that leverages memory re-sources effectively rather than minimally.

Besides, analysis of parameter count versus performance (**Figure 5D**) reveals that MambaVision exploits moderate-to-large model capacity to achieve superior representation quality. Although Transform-er-based models attain reasonable performance with fewer parameters, their accuracy saturates earlier. CNN-based models remain highly compact but struggle to model complex visual patterns, underscoring intrinsic limitations of convolutional inductive bias. Importantly, pre-training improves performance without increasing parameter count, confirming that representation quality—not model size alone—drives the observed gains.

Taken together, these results demonstrate that SSM-based and hybrid architectures, exemplified by MambaVision, achieve the most favorable balance between performance and efficiency. Compared with Transformer-based models, MambaVision consistently attains higher, near-saturated F1-scores while incurring only moderate increases in Flops, memory usage, and parameter count, and while maintaining competitive FPS. Relative to CNN-based models, MambaVision delivers substantial performance improvements (approximately 10–30% in F1-score) at a computational cost that remains well within practical deployment limits.

### 4.7 Classification performance from qualitative perspectives

To further analyze feature learning behavior, we apply t-Distributed Stochastic Neighbor Embedding (t-SNE) to the final-layer representations of models trained under a moderate data regime (30% of the training set). The two-dimensional projections provide qualitative insights into class separability, representation compactness, and robustness to data im-balance.

**Figure 6:**
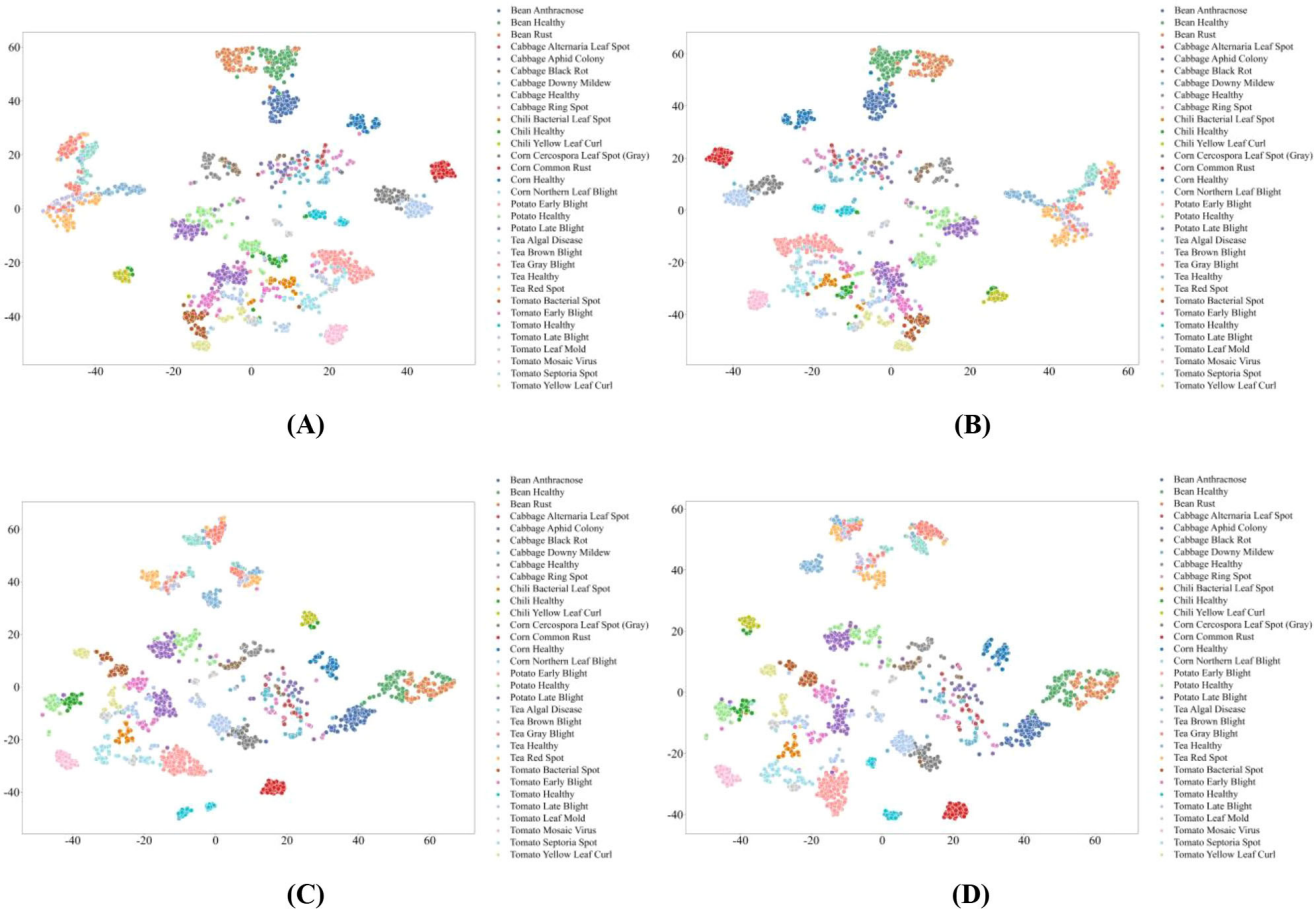
t-SNE Visualization of Feature Representations in MambaVision Models. **(A)** MambaVision-B; **(B)** MambaVision-B-21K; **(C)** MambaVision-L; **(D)** MambaVision-L-21K.

**Figure 7:**
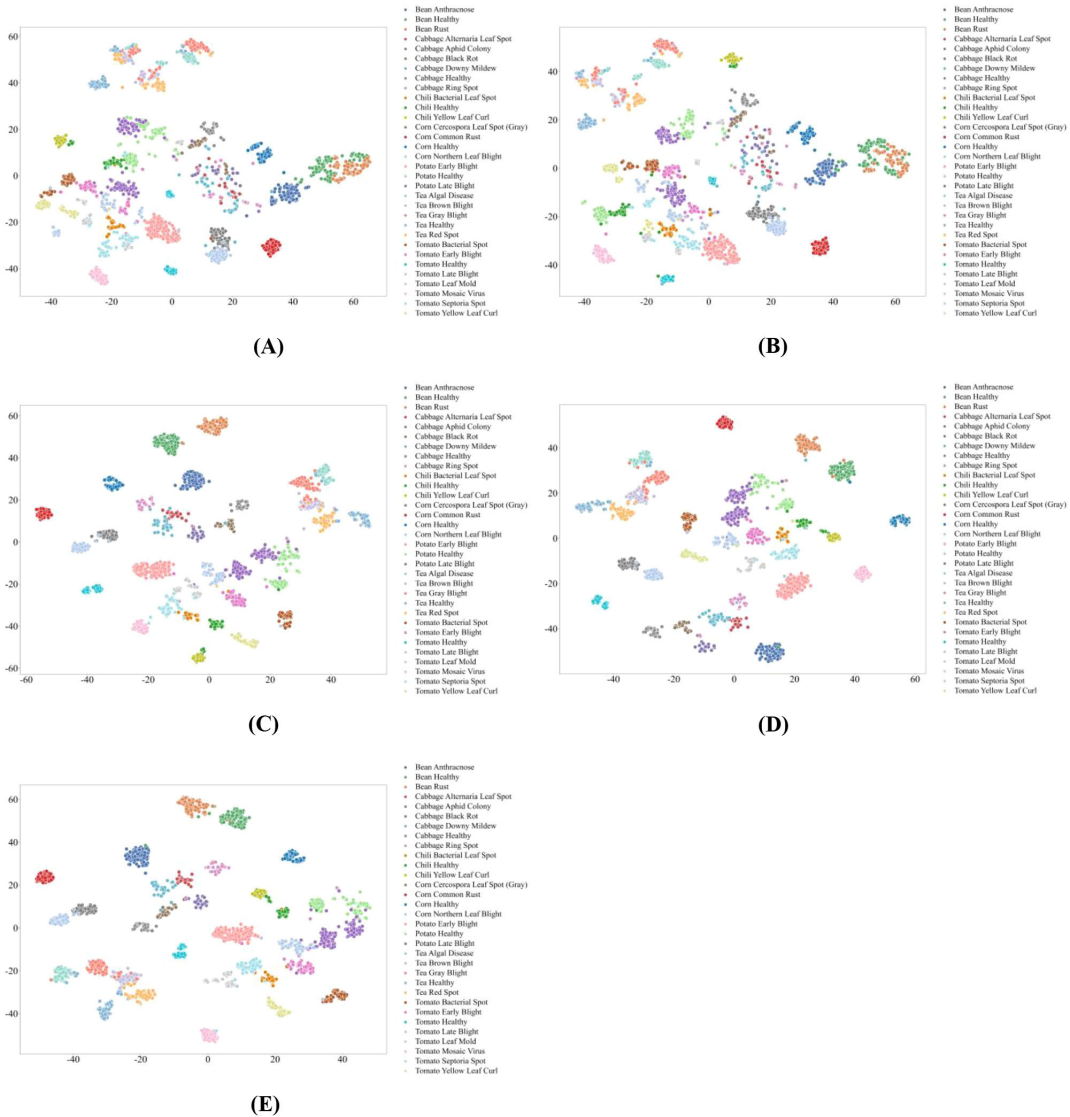
t-SNE Visualization of Feature Representations in MambaVision Models. **(A)** MambaVision-L2; **(B)** MambaVision-L2-512-21K; **(C)** MambaVision-S; **(D)** MambaVision-T; **(E)** MambaVision-T2.

Across MambaVision variants, feature representation quality exhibits clear capacity-dependent pat-terns. As shown in **Figures 6**, **7**, smaller variants form compact and well-separated clusters for most disease categories, including those with limited training samples, indicating concise and discriminative feature embeddings. Overlap among visually similar diseases is relatively limited, and healthy and diseased samples of the same crop are clearly separated. This behavior suggests that smaller models possess inductive biases well aligned with plant disease recognition, where localized texture cues dominate and excessive feature entanglement is avoided. The presence of multiple stable local clusters further indicates improved robustness for low-sample classes.

Intermediate and larger MambaVision variants achieve good separability for high-sample categories, while low-sample disease classes exhibit increased dispersion, as shown in **Figures 6**, **7**. Pretraining provides modest improvements in cluster compactness, primarily under data-sufficient conditions, without substantially altering representation structure. In contrast, variants with insufficient effective capacity or aggressive resolution scaling display fragmented and overlapping clusters, particularly among visually similar diseases and between healthy and diseased samples within the same crop. Higher input resolution amplifies background textures and illumination variations, which dilute disease-specific cues and counteract potential gains from increased model size or pretraining. These observations indicate that effective capacity allocation and feature focus are more critical than model scale alone for robust disease phenotyping.

**Figure 8:**
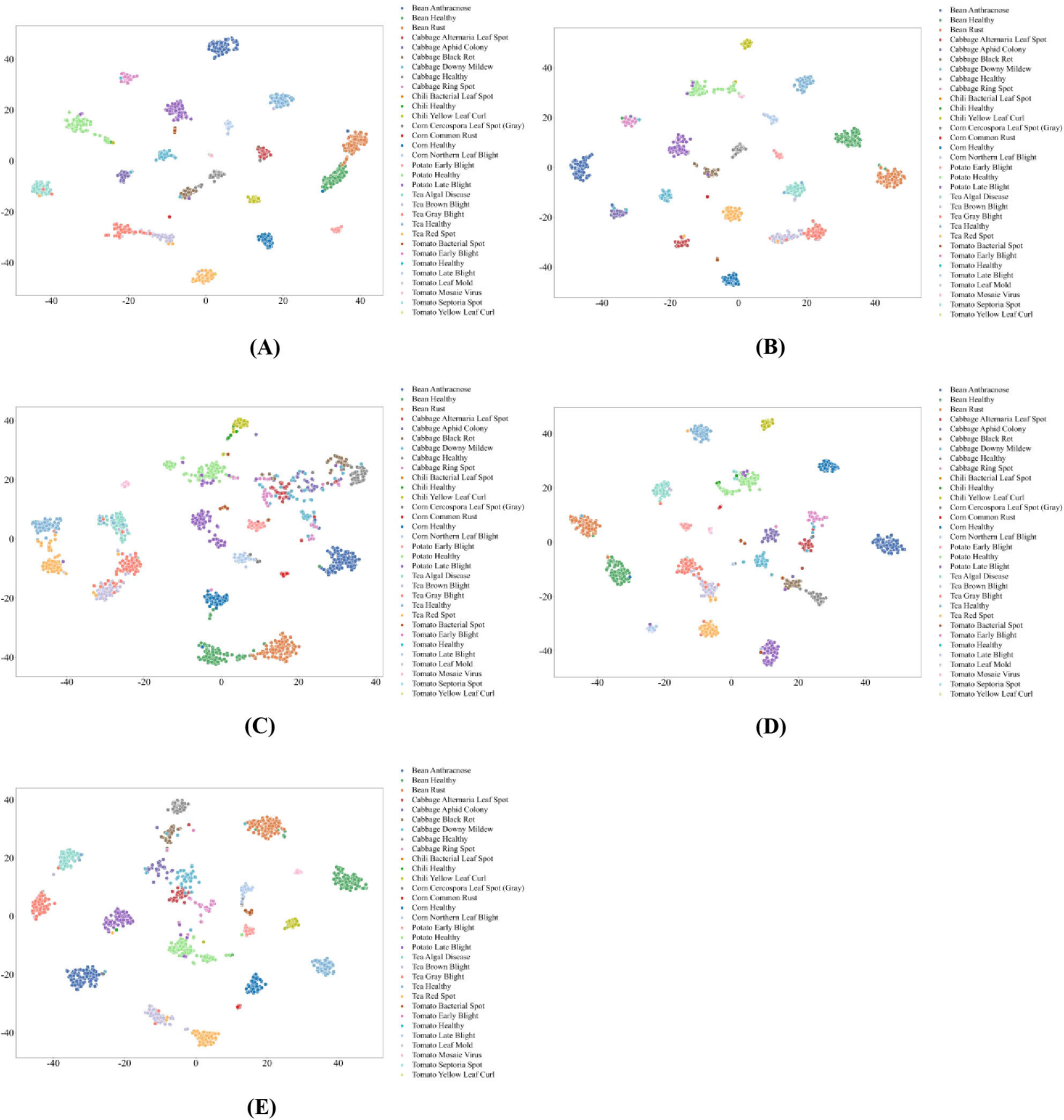
t-SNE Visualization of Feature Representations in Transformer-based Models. **(A)** CrossViT; **(B)** DeiT; **(C)** Focal Transformer; **(D)** PVT-Small; **(E)** Twins-SVT.

Transformer-based models show intermediate representation behavior, as shown in **Figure 8**. Architectures incorporating explicit multi-scale attention mechanisms produce relatively compact clusters with low intra-class variance and limited inter-class overlap, reflecting strong discriminative capability across disease categories. However, models relying on single-scale or localized attention exhibit increased dispersion and overlap, especially for visually similar diseases and low-sample categories. This pattern highlights a trade-off between global contextual modeling and robustness under data scarcity in Transform-er-based representations.

**Figure 9:**
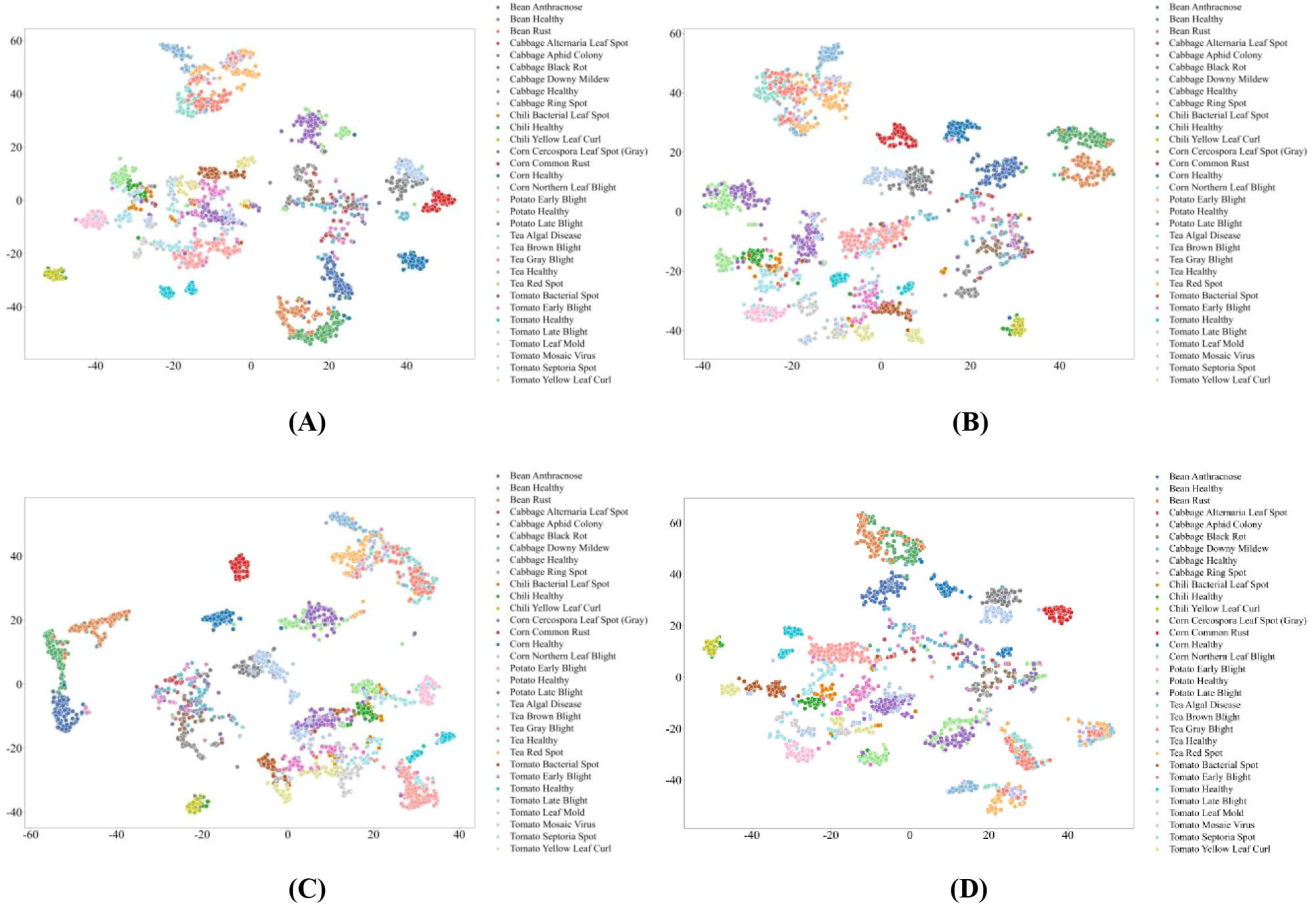
t-SNE Visualization of Feature Representations in CNN-based Models. **(A)** DenseNet; **(B)** ResNet; **(C)** ResNet_PreAct; **(D)** ResNeXt.

**Figure 10:**
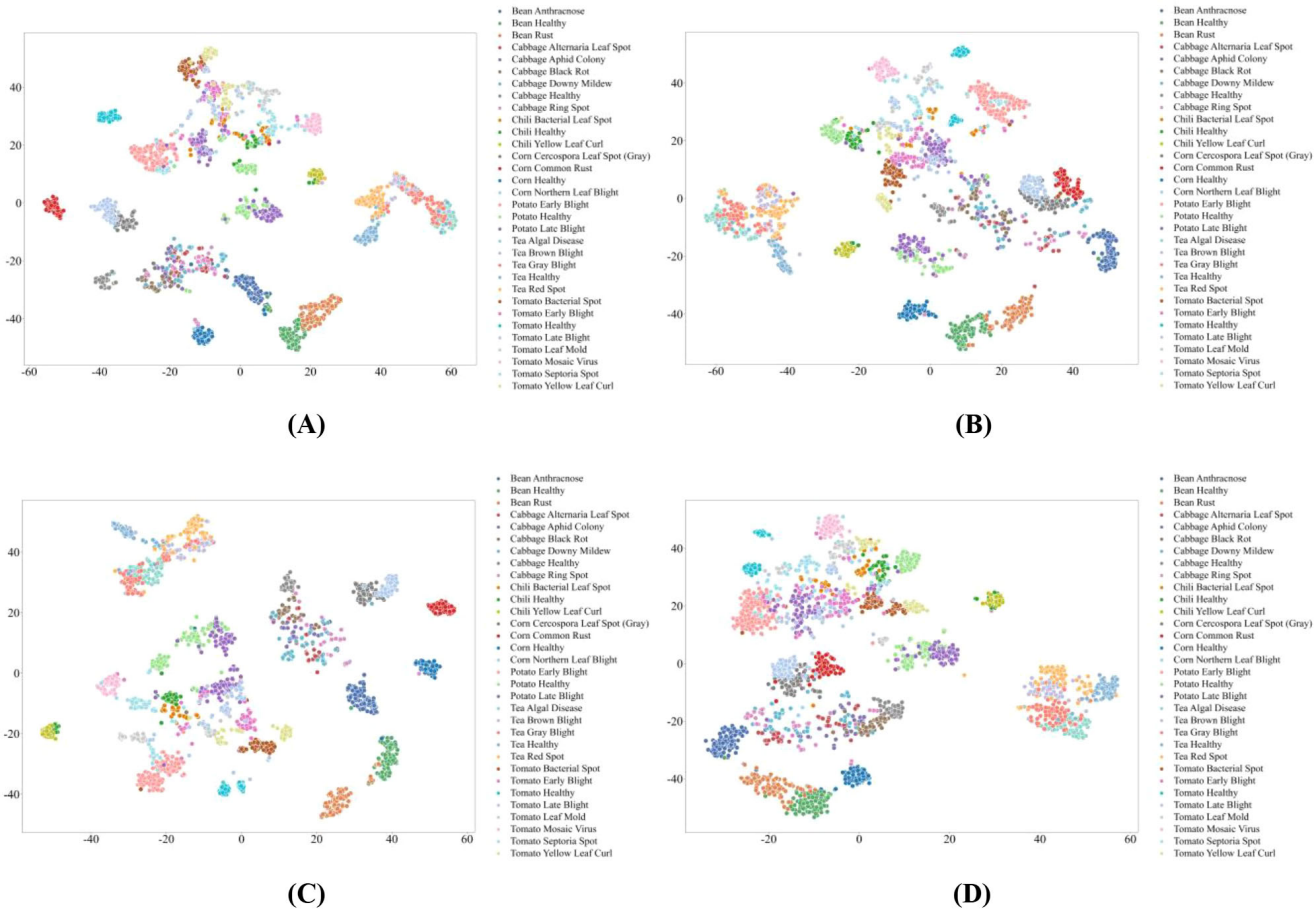
t-SNE Visualization of Feature Representations in CNN-based Models. **(A)** SE-ResNet-PReAct; **(B)** Shake-Shake; **(C)** WRN; **(D)** VGG.

CNN-based models predominantly rely on localized texture and shape cues, resulting in effective separation between healthy and diseased samples within the same crop, as shown in **Figures 9**, **10**. Nevertheless, their ability to discriminate visually similar diseases across different crops remains limited. Architectures enhanced with residual connections, channel attention, or widened feature maps yield more compact clusters and clearer class boundaries, whereas models with weaker feature reuse or aggregation mechanisms show widespread dispersion and substantial inter-class overlap. Low-sample disease categories are particularly prone to fragmented clustering, indicating limited generalization for fine-grained disease phenotypes.

Overall, the t-SNE analysis demonstrates that feature separability in plant pest and disease recognition is strongly governed by architectural mechanisms that balance local lesion characterization with global spatial context modeling. SSM-based MambaVision achieves the most consistent robustness across sample regimes, Transformer-based models provide effective but data-sensitive global representations, and CNN-based architectures remain constrained in distinguishing visually similar disease phenotypes under realistic agricultural conditions.

### 4.8 Per-class performance analysis on minority classes

To rigorously evaluate model robustness under data-scarcity conditions, we identified the six most under-represented categories within the 30% training subset. Each of these categories contains 140 training images, which corresponds to the minimum sample size across the dataset. The test set includes 60 images per class, ensuring unbiased evaluation. The selected categories are: cabbage_alternaria_leaf_spot, cabbage_aphid_colony, cabbage_black_rot, cabbage_downy_mildew, cabbage_healthy, and cabbage_ring_spot. We conducted a comparative analysis across MambaVision architectures, Transformer models, and CNN backbones, with detailed per-class evaluation.

MambaVision-based models demonstrate consistently strong and stable performance across all minority classes, even under limited training data (**Table 8**). For cabbage_aphid_colony and cabbage_healthy, all evaluated MambaVision variants achieve perfect classification (Recall = 1.0, Precision = 1.0, F1-score = 1.0), with no misclassified samples. For cabbage_alternaria_leaf_spot, cabbage_black_rot, and cabbage_ring_spot, F1-scores exceed 0.967 across all variants, with at most two misclassifications per class. Even for the most challenging category, cabbage_downy_mildew, the lowest F1-score remains as high as 0.898, indicating strong discriminative capability under limited data conditions.

**Table 8A:** Class-wise performance evaluation of MambaVision architectures on the cabbage disease dataset.

Model variant	Class name	Correctly predicted	Misclassified	Recall	Precision	F1-score
MambaVision-B	cabbage_alternaria_leaf_spot	60	0	100%	96.8%	98.4%
	cabbage_aphid_colony	60	0	100%	100%	100%
	cabbage_black_rot	58	2	96.7%	98.3%	97.5%
	cabbage_downy_mildew	55	5	91.7%	90.2%	90.9%
	cabbage_healthy	60	0	100.0%	98.4%	99.2%
	cabbage_ring_spot	58	2	96.7%	96.7%	96.7%
MambaVision-B-21K	cabbage_alternaria_leaf_spot	60	0	100%	95.2%	97.6%
	cabbage_aphid_colony	60	0	100%	100%	100%
	cabbage_black_rot	57	3	95.0%	96.6%	95.8%
	cabbage_downy_mildew	53	7	88.3%	88.3%	88.3%
	cabbage_healthy	59	1	98.3%	98.3%	98.3%
	cabbage_ring_spot	58	2	96.7%	96.7%	96.7%
MambaVision-L	cabbage_alternaria_leaf_spot	58	2	96.7%	98.3%	97.5%
	cabbage_aphid_colony	60	0	100%	100%	100%
	cabbage_black_rot	59	1	98.3%	98.3%	98.3%
	cabbage_downy_mildew	57	3	95%	93.4%	94.2%
	cabbage_healthy	60	0	100%	98.4%	99.2%
	cabbage_ring_spot	59	1	98.3%	98.3%	98.3%
MambaVision-L-21K	cabbage_alternaria_leaf_spot	60	0	100%	100.0%	100%
	cabbage_aphid_colony	60	0	100%	100.0%	100%
	cabbage_black_rot	59	1	98.3%	98.3%	98.3%
	cabbage_downy_mildew	56	4	93.3%	90.3%	91.8%
	cabbage_healthy	60	0	100%	98.4%	99.2%
	cabbage_ring_spot	59	1	98.3%	98.3%	98.3%

**Table 8B:** Class-wise performance evaluation of MambaVision architectures on the cabbage disease dataset.

Model variant	Class name	Correctly predicted	Misclassified	Recall	Precision	F1-score
MambaVision-T	cabbage_alternaria_leaf_spot	58	2	96.7%	96.7%	96.7%
	cabbage_aphid_colony	59	1	98.3%	98.3%	98.3%
	cabbage_black_rot	60	0	100%	96.8%	98.4%
	cabbage_downy_mildew	58	2	96.7%	95.1%	95.9%
	cabbage_healthy	60	0	100.0%	96.8%	98.4%
	cabbage_ring_spot	59	1	98.3%	98.3%	98.3%
MambaVision-T2	cabbage_alternaria_leaf_spot	58	2	96.7%	96.7%	96.7%
	cabbage_aphid_colony	60	0	100%	98.4%	99.2%
	cabbage_black_rot	59	1	98.3%	98.3%	98.3%
	cabbage_downy_mildew	56	4	93.3%	90.3%	91.8%
	cabbage_healthy	60	0	100.0%	98.4%	99.2%
	cabbage_ring_spot	59	1	98.3%	98.3%	98.3%
MambaVision-S	cabbage_alternaria_leaf_spot	58	2	96.7%	96.7%	96.7%
	cabbage_aphid_colony	60	0	100.0%	96.8%	98.4%
	cabbage_black_rot	58	2	96.7%	95.1%	95.9%
	cabbage_downy_mildew	59	1	98.3%	98.3%	98.3%
	cabbage_healthy	60	0	100%	96.8%	98.4%
	cabbage_ring_spot	60	0	100%	98.4%	99.2%
MambaVision-L2	cabbage_alternaria_leaf_spot	60	0	100%	98.4%	99.2%
	cabbage_aphid_colony	60	0	100%	100%	100%
	cabbage_black_rot	59	1	98.3%	98.3%	98.3%
	cabbage_downy_mildew	56	4	93.3%	93.3%	93.3%
	cabbage_healthy	60	0	100.0%	98.4%	99.2%
	cabbage_ring_spot	59	1	98.3%	98.3%	98.3%
MambaVision-L2-512-21K	cabbage_alternaria_leaf_spot	59	1	98.3%	96.7%	97.5%
	cabbage_aphid_colony	60	0	100%	100%	100%
	cabbage_black_rot	60	0	100%	98.4%	99.2%
	cabbage_downy_mildew	53	7	88.3%	91.4%	89.8%
	cabbage_healthy	60	0	100%	100%	100%
	cabbage_ring_spot	59	1	98.3%	98.3%	98.3%

Transformer-based models (e.g., CrossViT, DeiT, and Twins) also exhibit relatively stable performance on these minority classes (**Table 9**). For instance, CrossViT achieves F1-scores above 0.918 across all six categories, while DeiT maintains F1-scores above 0.898. Although the Focal Transformer shows noticeable performance degradation, this appears to be related to its specific architectural design rather than a general limitation of Transformer-based approaches.

**Table 9:** Comparative analysis of Transformer-based models for cabbage disease recognition.

Network architecture	Disease and pest categories	Correctly predicted	Misclassified	Recall	Precision	F1-score
CrossViT	cabbage_alternaria_leaf_spot	59	1	98.3%	98.3%	98.3%
	cabbage_aphid_colony	60	0	100.0%	96.8%	98.4%
	cabbage_black_rot	59	1	98.3%	95.2%	96.7%
	cabbage_downy_mildew	57	3	95%	90.5%	92.7%
	cabbage_healthy	56	4	93.3%	90.3%	91.8%
	cabbage_ring_spot	59	1	98.3%	96.7%	97.5%
Twins	cabbage_alternaria_leaf_spot	57	3	95%	96.6%	95.8%
	cabbage_aphid_colony	54	6	90%	91.5%	90.8%
	cabbage_black_rot	60	0	100%	96.8%	98.4%
	cabbage_downy_mildew	52	8	86.7%	85.2%	86%
	cabbage_healthy	60	0	100%	96.8%	98.4%
	cabbage_ring_spot	60	0	100%	95.2%	97.6%
DeiT	cabbage_alternaria_leaf_spot	59	1	98.3%	98.3%	98.3%
	cabbage_aphid_colony	59	1	98.3%	96.7%	97.5%
	cabbage_black_rot	56	4	93.3%	96.6%	94.9%
	cabbage_downy_mildew	56	4	93.3%	90.3%	91.8%
	cabbage_healthy	53	7	88.3%	91.4%	89.8%
	cabbage_ring_spot	59	1	98.3%	96.7%	97.5%
Pvt Small	cabbage_alternaria_leaf_spot	59	1	98.3%	98.3%	98.3%
	cabbage_aphid_colony	57	3	95%	93.4%	94.2%
	cabbage_black_rot	59	1	98.3%	85.5%	91.5%
	cabbage_downy_mildew	50	10	83.3%	79.4%	81.3%
	cabbage_healthy	49	11	81.7%	78.4%	80%
	cabbage_ring_spot	60	0	100%	92.3%	96%
Focal Transformer	cabbage_alternaria_leaf_spot	55	5	91.7%	79.7%	85.3%
	cabbage_aphid_colony	54	6	90%	77.1%	83.1%
	cabbage_black_rot	47	13	78.3%	74.6%	76.4%
	cabbage_downy_mildew	39	21	65.0%	58.2%	61.4%
	cabbage_healthy	55	5	91.7%	90.2%	90.9%
	cabbage_ring_spot	23	37	38.3%	30.7%	34.1%

In contrast, conventional CNN models show more pronounced performance variability on minority classes (**Table 10**). For example, the lowest F1-score observed for ResNet_PReAct drops to 0.248, indicating substantial difficulty in learning discriminative features under limited data conditions (**Table 10**).

**Table 10:** Benchmarking results of conventional CNN backbones on cabbage leaf disease classification.

Network architecture	Disease and pest categories	Correctly predicted	Misclassified	Recall	Precision	F1-score
Shake-Shake	cabbage_alternaria_leaf_spot	55	5	91.7%	91.7%	91.7%
	cabbage_aphid_colony	59	1	98.3%	96.7%	97.5%
	cabbage_black_rot	45	15	75%	80.4%	77.6%
	cabbage_downy_mildew	33	27	55%	63.5%	58.9%
	cabbage_healthy	55	5	91.7%	93.2%	92.4%
	cabbage_ring_spot	56	4	93.3%	87.5%	90.3%
VGG	cabbage_alternaria_leaf_spot	51	9	85%	89.5%	87.2%
	cabbage_aphid_colony	57	3	95%	90.5%	92.7%
	cabbage_black_rot	53	7	88.3%	91.4%	89.8%
	cabbage_downy_mildew	43	17	71.7%	75.4%	73.5%
	cabbage_healthy	59	1	98.3%	95.2%	96.7%
	cabbage_ring_spot	47	13	78.3%	77%	77.7%
WRN	cabbage_alternaria_leaf_spot	45	15	75%	76.3%	75.6%
	cabbage_aphid_colony	56	4	93.3%	90.3%	91.8%
	cabbage_black_rot	43	17	71.7%	75.4%	73.5%
	cabbage_downy_mildew	25	35	41.7%	48.1%	44.6%
	cabbage_healthy	56	4	93.3%	90.3%	91.8%
	cabbage_ring_spot	47	13	78.3%	79.70%	79%
ResNet	cabbage_alternaria_leaf_spot	32	28	53.3%	52.50%	52.9%
	cabbage_aphid_colony	38	22	63.3%	55.9%	59.4%
	cabbage_black_rot	52	8	86.7%	85.2%	86%
	cabbage_downy_mildew	29	31	48.3%	53.7%	50.9%
	cabbage_healthy	55	5	91.7%	87.3%	89.4%
	cabbage_ring_spot	38	22	63.3%	53.5%	58%
	cabbage_ring_spot	20	40	33.3%	35.1%	34.2%

**Table 10B:** Benchmarking results of conventional CNN backbones on cabbage leaf disease classification.

Network architecture	Disease and pest categories	Correctly predicted	Misclassified	Recall	Precision	F1-score
DenseNet	cabbage_alternaria_leaf_spot	46	14	76.7%	73%	74.8%
	cabbage_aphid_colony	50	10	83.3%	78.1%	80.6%
	cabbage_black_rot	46	14	76.7%	70.8%	73.6%
	cabbage_downy_mildew	38	22	63.3%	64.4%	63.8%
	cabbage_healthy	56	4	93.3%	80.%	86.2%
	cabbage_ring_spot	47	13	78.3%	72.3%	75.2%
SE-ResNet-PReAct	cabbage_alternaria_leaf_spot	39	21	65%	66.1%	65.5%
	cabbage_aphid_colony	31	29	51.70%	56.4%	53.9%
	cabbage_black_rot	53	7	88.30%	79.1%	83.5%
	cabbage_downy_mildew	27	33	45%	51%	47.8%
	cabbage_healthy	60	0	100%	85.7%	92.3%
	cabbage_ring_spot	38	22	63.3%	59.4%	61.3%
ResNeXt	cabbage_alternaria_leaf_spot	40	20	66.7%	83.3%	74.1%
	cabbage_aphid_colony	57	3	95%	95%	95%
	cabbage_black_rot	19	41	31.7%	54.3%	40%
	cabbage_downy_mildew	28	32	46.7%	62.2%	53.3%
	cabbage_healthy	47	13	78.3%	77.%	77.7%
	cabbage_ring_spot	42	18	70%	76.4%	73%
ResNet_PReAct	cabbage_alternaria_leaf_spot	29	31	48.3%	46%	47.1%
	cabbage_aphid_colony	19	41	31.7%	33.90%	32.8%
	cabbage_black_rot	37	23	61.7%	52.9%	56.9%
	cabbage_downy_mildew	15	45	25.%	24.6%	24.8%
	cabbage_healthy	53	7	88.3%	76.8%	82.1%

Overall, the per-class analysis indicates that, even for categories with minimal training samples, modern architectures—particularly MambaVision and Transformer-based models—can achieve high and stable performance in this closed-set fine-grained disease classification task. These findings suggest that model architecture plays a critical role in handling visually similar categories under limited data regimes, and support the reliability of the comparative conclusions presented in this study.”

## 5 Discussions

### 5.1 Symptom-type dependency of model paradigms: local texture versus spatial organization

In this work, we reorganized the classification results by grouping the 32 disease categories according to symptom appearance (Spot, Necrosis, Surface-colonizing, Mosaic, Insect, and Healthy), and analyzed the group-wise average F1-scores under the 30% training regime.

As shown in the symptom-level comparison (**Table 6**), CNN-based models perform competitively on spot-type symptoms, where disease manifestation is dominated by localized, high-contrast lesions with clear boundaries. For these symptoms, strong local texture extraction is often sufficient, allowing CNN-based models to approach the performance of more advanced architectures. In contrast, their performance degrades markedly for necrosis and surface-colonizing symptoms, which involve lesion merging, diffuse expansion, and large-scale spatial organization across the leaf surface.

Transformer-based models demonstrate improved performance on symptom groups requiring global context modeling, such as mosaic and necrosis, but their results remain more variable across symptom types. In comparison, hybrid MambaVision-based models consistently achieve high and stable F1-scores across all symptom groups, particularly for complex phenotypes that require joint interpretation of fine-grained textures and long-range spatial distribution. These results show that paradigm-level performance differences are closely linked to symptom morphology and spatial organization rather than overall classification difficulty.

### 5.2 Data efficiency and performance degradation under extreme sample scarcity

Under the most extreme setting (1% training data), hybrid MambaVision-based models retain approximately 60–80% accuracy, whereas CNN-based models experience severe degradation, often falling below 30%, and transformer-based models show larger fluctuations across architectures.

This behavior suggests improved data-efficiency characteristics. Rather than claiming absolute superiority, our analysis shows that all architectural paradigms experience performance degradation under limited data. The key distinction lies in the rate of degradation: hybrid MambaVision-based models degrade more gracefully, retaining a larger proportion of their full-data performance as training samples decrease. This relative performance retention is more practically meaningful than peak accuracy alone, particularly in real agricultural scenarios where labeled data is limited. Importantly, all comparisons are conducted under identical experimental settings on the same integrated dataset, ensuring fair cross-paradigm evaluation.

From a phenotyping perspective, this robustness is critical. Agricultural disease datasets are often imbalanced and difficult to annotate, particularly for rare or early-stage symptoms. The ability of hybrid MambaVision-based models to preserve discriminative representations under limited data suggests that hybrid local–global modeling helps mitigate overfitting to spurious textures while still capturing essential spatial cues.

### 5.3 Global modeling strength versus instability of transformers under field complexity

Despite strong global modeling capacity, Transformer-based models exhibit greater instability in small-sample regimes. Our results show that when training data are limited (≤10%), Transformer-based models display larger performance variance across runs and symptom groups compared with hybrid MambaVision-based models, even though they often outperform CNN-based models on average.

This phenomenon can be explained by the interaction between field complexity and attention-based global modeling. Agricultural images captured under natural conditions exhibit heterogeneous backgrounds, illumination variation, and non-disease visual patterns. Transformer-based models, which rely heavily on global attention, are more sensitive to data quantity and regularization strength; insufficient data may cause attention mechanisms to amplify irrelevant global correlations rather than disease-specific cues.

In contrast, hybrid MambaVision-based models constrain global dependency modeling through state-space dynamics while preserving convolutional inductive bias for local textures. This architectural balance reduces sensitivity to data scarcity and stabilizes learning under realistic field conditions. Therefore, the observed instability of Transformer-based models under limited data does not contradict their modeling strength, but rather highlights a data-architecture interaction effect.

To summarize, these analyses demonstrate that paradigm-level differences in plant disease recognition are governed not only by model capacity, but by the alignment between architectural inductive bias, symptom morphology, and data availability, with hybrid MambaVision-based models providing the most robust compromise across these dimensions.

### 5.4 Limitations on comparison with prior work

A methodological limitation of this study concerns the comparison with prior work. A large body of existing research in plant disease classification reports performance on the PlantVillage dataset, which has become a widely used benchmark in this field. In Section 2, we cited representative studies (e.g., Mohanty et al., 2016; Ferentinos, 2018; Zhu et al., 2023a) to provide context for the development of deep learning approaches in this domain. However, direct numerical comparison between our results and those reported in prior PlantVillage-based studies is not strictly appropriate. This is due to several important differences: (1) our dataset is constructed by integrating multiple public sources, resulting in more diverse imaging conditions and class distributions; (2) our experiments are conducted under a unified and controlled training protocol, including standardized data splits, preprocessing, and evaluation metrics, which may differ from those used in prior studies; and (3) the 32-class taxonomy adopted in this work differs from the category definitions used in the PlantVillage dataset.

Therefore, the performance values reported in prior work are included for contextual reference only, rather than as a basis for direct quantitative comparison. This study does not aim to claim state-of-the-art performance against existing benchmarks. Instead, the primary contribution of this work lies in the systematic comparison of different model architectures under a unified experimental framework. By evaluating CNN-, Transformer-, and MambaVision-based models under identical conditions, this study provides reliable insights into their relative performance characteristics across varying data availability regimes. We encourage future work to adopt more diverse datasets and standardized evaluation protocols to facilitate more rigorous and meaningful cross-study comparisons in plant disease recognition.

## 6 Conclusion and summary

This study presents a unified, paradigm-level evaluation of CNN-, Transformer-, and hybrid MambaVision-based models for multi-crop pest and disease image classification under realistic agricultural conditions. Results reveal clear differences in data efficiency and robustness across architectures. CNN-based models are effective for diseases dominated by localized lesion textures but remain limited by restricted global context modeling. Transformer-based models benefit from global dependency modeling and generally outperform classical CNNs under moderate data availability, yet exhibit increased instability under severe data scarcity. In contrast, hybrid MambaVision-based models consistently achieve the most stable and accurate performance across all data regimes, particularly under limited training samples, by effectively integrating fine-grained lesion representation with efficient long-range spatial modeling. Feature visualization further confirms their robustness for low-sample and visually similar disease categories. Combined with favorable accuracy-efficiency trade-offs, these results indicate that MambaVision offers a practical and reliable solution for field-deployable crop pest and disease recognition. Future work will focus on extending paradigm-level analysis to earlier disease stages, cross-crop generalization, and multi-modal field data, aiming to further bridge deep learning-based diagnosis with practical plant disease phenotyping and precision crop management.

## Data Availability

The original contributions presented in the study are included in the article/supplementary material, further inquiries can be directed to the corresponding author/s.

## Footnotes

1 https://www.kaggle.com/datasets/silviarahma2702/plants-diseases and https://www.kaggle.com/datasets/mubbassir/balanced-cabbage-dataset-200-each were accessed on January 31, 2026.
